# Between love and pain: A qualitative analysis of menstrual distress and romantic relationships

**DOI:** 10.1111/aphw.70219

**Published:** 2026-09-23

**Authors:** Theresa J. S. Wallner, Nora Christlein, Laura M. König

**Affiliations:** ^1^ Department of Clinical and Health Psychology University of Vienna Vienna Austria; ^2^ Department of Psychology University of Konstanz Konstanz Germany

**Keywords:** couples, menstrual cycle, pain, psychological well‐being, social support

## Abstract

Menstrual distress ‐ the physiological and emotional stress response to menstrual symptoms that are perceived as threatening and difficult to manage ‐ is common among menstruating people and can have a profound negative impact on mental health and well‐being. Romantic partners can support dealing with emotional and physical stress, yet their role in dealing with menstrual distress specifically remains underexplored. Adopting a biopsychosocial perspective, this qualitative study aims to shed light on the experience of menstrual distress within romantic relationships. Semi‐structured interviews were conducted with 27 individuals (aged 19–59 years, living in Austria), who were in romantic relationships (*n* = 23 heterosexual, *n* = 3 homosexual/queer, *n =* 1 unlabelled) and who self‐reported to have experienced menstrual distress. Indeed, romantic partners play a crucial role in shaping both the impact of and coping mechanisms for menstrual distress. Due to the intimate nature of romantic relationships, partners influence experiences of menstrual distress, whether actively engaging with the topic or not. Supportive relationships fostering authenticity, communication and teamwork enhance coping, whereas negative partner responses can perpetuate negative stereotypes, stigma and symptom trivialisation. To enhance individuals' and couples' well‐being, educational campaigns should raise menstrual health awareness early, and couples may benefit from guidance in managing the challenges effectively.

## INTRODUCTION

Menstruating people^1^ experience cyclical hormonal changes (i.e., rise and fall of the follicle‐stimulating hormone, luteinizing hormone, oestrogen and progesterone; Chrisler, 2008) that cause diverse physiological, behavioural and emotional changes across the different cycle phases (i.e., ovulation, luteal phase, menstruation; Clayton, 2008; Negriff et al., 2009). These changes are referred to as menstrual symptoms. Common menstrual symptoms include pain and cramps in different areas, gastrointestinal changes, changes in physiological functions (e.g., appetite, sleep and sexual drive), as well as affective and cognitive changes (e.g., sadness, irritability, anxiety and concentration; Vannuccini et al., 2021). Menstrual symptoms are common among menstruating people (cf., Volpi & König, 2025). For example, almost 70% of menstruators experience medium to strong menstrual pain (Gaiswinkler et al., 2024), 85% of menstruators experience abdominal pain during their period, 53% experience heavy bleeding and more than 70% cognitive and affective symptoms as well as tiredness (Schoep et al., 2019). Menstrual symptoms can be part of the typical menstrual experience, but they can also be caused by underlying menstrual disorders like endometriosis, where endometrial‐like tissue is present outside the uterus’ causing pain, cysts and adhesions (Culley et al., 2017, p. 1168).

Both clinical and non‐clinical menstrual symptoms can significantly impair daily life and cause menstrual distress. In the survey by Schoep et al. (2019), 38% of women reported that menstrual symptoms limited their daily activities, with some experiencing severe restrictions in daily functioning. Thus, menstrual symptoms can have a negative impact on physiological, mental and emotional well‐being (Matteson et al., 2013) as well as affect quality of life (Sarita et al., 2024).

Menstrual distress describes the physiological and emotional response to the experience of menstrual symptoms, if they are appraised as threatening to the individual (Vannuccini et al., 2021), for example, if there is a perceived inability to cope with available resources (see also Ridner, 2004). In other words, two menstruating people with the same menstrual symptoms might appraise menstrual symptoms differently due to different daily demands, resources for support and locus of control, and thus experience different levels of menstrual distress. Hence, not every menstrual symptom necessarily leads to menstrual distress and not all menstrual distress is pathological. For example, Schick et al. (2022) found that the stage of endometriosis did not correlate significantly with the distress score of patients. Consequently, the experience of menstrual distress can be independent of prevalent menstrual disorders. This finding demonstrates that other aspects than mere biological symptoms are relevant for the experience of menstrual distress.

In this context, romantic relationships may play a critical role (cf., Schick et al., 2022), given the amount of time spent with a romantic partner and higher intimacy levels in communication compared with friends, colleagues or other social contacts. For example, during phases with high menstrual distress, an affected person might call in sick at work and refrain from social interaction with friends but might more likely or even have to still communicate with their partner (e.g., in case of living together). Aside from direct communication, daily activities and sexual intimacy may also change along with menstrual distress. Indeed, social relationships are crucial for health and well‐being in general and romantic partners can directly and indirectly influence health outcomes (Kiecolt‐Glaser & Newton, 2001). For example, Fordyce (1976) pointed out the role of romantic partners in perpetuating chronic pain. Using the operant model, he demonstrated that the partner's response to pain behaviour reinforced or reduced those behaviours through operant learning. Since then, complex models have been introduced to explain the role of partners in dealing with chronic pain, but the partner's response remains crucial for coping. As chronic pain symptoms and menstrual symptoms can overlap (e.g., lower back pain, pelvic pain and vulvovaginal pain) and similarly impact an individual's' daily functioning, these insights might be transferable to coping with menstrual symptoms.

Despite the potentially high relevance of romantic partners in coping with menstrual distress, research at this interface is still limited. For instance, best‐practice guidelines for the management of endometriosis evaluate surgical and pharmacological treatment such as hormonal therapy but do not mention the role of romantic partners in managing impairment in daily and social life (Becker et al., 2022; Kalaitzopoulos et al., 2021). More recently, studies on menstrual disorders started to acknowledge the relevance of romantic partners, who were perceived as highly relevant source of support by women suffering from endometriosis, but some of them also mentioned that their menstrual disorder strained both their partners and their romantic relationships, ultimately leading to break‐ups (Denny, 2004). Moreover, romantic partners of women suffering from endometriosis reported increased financial worries, restrictions in daily activities, feeling burdened by additional tasks, affected sex life and feeling emotionally strained (Culley et al., 2017). These studies demonstrate a mutual influence of romantic relationships and menstrual distress. Yet, a deeper understanding of this interplay is lacking, especially in individuals not (yet) diagnosed with menstrual disorders.

In the present study, we address this gap and adopt a biopsychosocial perspective (introduced by Engel, 1977) on menstrual distress. We emphasise that menstruation and menstrual symptoms are not purely a biological phenomenon but must be viewed in a social and psychological context to understand the experience of menstrual distress (Chrisler, 2008; Johnston‐Robledo & Stubbs, 2013). Regarding the social context, menstruation and menstrual symptoms are shaped by historical and socio‐cultural influences, including stigmatisation, social norms and trivialisation. For example, menstruation has historically been linked to fear, disgust and shame across cultures and religions, thereby reinforcing stigma and taboo around menstruation (Roberts et al., 2002). Importantly, resulting social norms persist to this day and manifest themselves not least in concealment (cf., Wood, 2020), that is, the expectation to hide menstrual symptoms (Kissling, 1996). Indeed, menstrual distress is often trivialised by menstruating people and medical staff (Clark, 2012; Denny, 2004; Li et al., 2020), leading to the belief that pain during menstruation is normal and should be endured (Gaiswinkler et al., 2024). If menstruating people are expected to hide their menstruation, worries about visible leaking and odour (cf., O'Flynn, 2006), the fear or actual experience of being seen as overdramatic when disclosing menstrual symptoms and internalised stigma further increase menstrual distress. Inevitably, concealment, stigma and taboo have consequences for the appraisal of menstrual symptoms, both inside and outside romantic relationships, highlighting the psychological dimension of the biopsychosocial approach.

This study aimed to provide deeper insights into the role of the romantic partner in coping with menstrual distress, covering a broad range of menstrual symptoms and varying levels of menstrual distress. Importantly, we considered bidirectional processes of support and strain, that is, effects of romantic relationships on dealing with menstrual distress and effect of experiencing menstrual distress on romantic relationships. Through an in‐depth qualitative research approach that includes romantic relationship dynamics, it is possible to contextualise menstrual distress within a biopsychosocial framework.

## MATERIALS AND METHODS

The study received ethical approval by the local university's ethics committee and was carried out in accordance with the Declaration of Helsinki. Because the study took place in Austria, interviews were conducted in German. Any quotes included in the manuscript to illustrate the findings were translated into English and checked by a second researcher for consistency of meaning. Study materials are openly available on the Open Science Framework (OSF; https://osf.io/vu5dg/).

### Materials

The study material comprised a questionnaire and the interview guide. The questionnaire contained items on demographics (age, gender, occupation and sexual orientation), participants' relationship (duration, type,^2^ shared household and childcare) and menstruation experiences (period in the past 12 months, menstruation‐related diagnoses and related medication). In addition, relationship satisfaction (defined as general satisfaction with the romantic relationship; ‘How satisfied are you in your relationship?’) and relationship happiness (defined as perceived happiness in the relationship; ‘How happy are you in your relationship?’) were assessed on a 7‐point Likert scale (ranging from 1 [*not at all*] to 7 [*extremely*]) based on Fletcher et al. (2000). Furthermore, the Menstrual Distress Questionnaire (MEDI‐Q) by Vannuccini et al. (2021) was included, containing 25 menstrual symptoms (physical, cognitive and emotional), asking for each whether symptoms occurred during the menstrual phase on a 3‐point scale (ranging from *yes, more than half of the times I've had my period* to *no, the symptom did not occur*) and whether symptoms interfered with quality of life during the menstrual phase, the premenstrual phase and the intermenstrual phase. To assess impairment, a 5‐point Likert scale was used (ranging from *not all* to *very much* and *never had this symptom*). The MEDI‐Q measures the number of menstrual symptoms that generate menstrual distress (not every symptom creates distress), the average and global menstrual distress and the specificity of menstrual distress (i.e., the proportion of symptoms that generate more distress during menstruation compared with other menstrual phases). The MEDI‐Q provided background information during interviews so that the interviewer could refer back to low vs high experienced distress when menstruating and scores were used to contextualise interview excerpts.

The design of the semi‐structured interview guide, the elaboration of the interview questions and data analysis followed the seven stages to conduct interviews by Kvale (1996). These involve thematising, designing, interviewing, transcribing, analysing, verifying and reporting. The interview questions were obtained through literature research on related topics and through own ideas, refined in discussions between the authors. The draft of the manual was further discussed with experts in qualitative research and refined afterwards. In addition, test interviews were conducted using a preliminary version of the interview guide and feedback was incorporated into the final interview guide. Due to changes made between the preliminary and final interview guide, the test interviews were not included in the analysis.

### Data collection

The interviews took place in Austria in summer 2024 on weekdays in the facilities of the study team at their university. Participants were recruited by distributing flyers in public spaces and the university. In addition, the study was advertised on social media, on the university's platform for studies and via a university mailing list. Strategic distribution of the flyers, including at cafés with different target customers, women's centres, and online communities for queer people aimed to achieve a sample diverse in the aspects of menstrual symptoms, age and types and lengths of relationships.

The interviews lasted 40 min on average (range 25–60 min). All interviews were conducted by the same female researcher. No prior personal connection had been established between the interviewer and interviewees except for one case.^3^ As most participants were in a similar life phase as the interviewer and both parties shared experiences related to menstrual distress, rapport was easily built with most of the participants (i.e., this setting allowed for a sense of ‘womanhood’ or ‘menstruation‐hood’; cf., similar experiences; Brantelid et al., 2014). Rapport is essential for qualitative interviews (Kvale & Brinkmann, 2009) and seen as a prerequisite for building trust, thus allowing interviewees to share personal experiences.

Upon arrival, participants were first welcomed, with the interviewer introducing herself and offering to be on first‐name basis, which is not common in German‐speaking countries but should have allowed for a more personal atmosphere. The interviewer and the interviewee sat diagonally across each other with a table in between and the chairs positioned in a way that direct eye contact was not forced. The interviewer explained the study procedure before the interviewee signed the informed consent form. Next, participants completed the pen‐and‐paper questionnaire. The interviewer then screened the answers of the questionnaire to learn about the interviewee's romantic relationship and main menstrual symptoms. Only then the audio recording started.

The interview questions were all open‐ended and divided into three blocks, namely, menstrual distress, the romantic relationship and the link between menstrual distress and the romantic relationship. The interview guide including the interview questions is available on OSF (https://osf.io/vu5dg/). The order of the first two blocks was varied depending on what the interviewee first mentioned as motivation to participate in the study. For example, if a participant stated that they were drawn to the study because they experience heavy menstrual symptoms, the interviewer started with the block on menstrual symptoms before moving to the other two blocks. The number of questions, including follow‐up questions, depended on the talkativeness of the interviewees. If necessary, further questions were asked for clarification. Silence was embraced to give interviewees time to think and answer. The interview session ended with a positive outlook, asking interviewees what they wished for future coping with menstrual distress.

After the interview, the recording was stopped; the interviewees were debriefed and offered an information sheet providing topic‐related resources such as recommended literature and local institutions for further information and counselling. For compensation, participants received €15 or 6 study participation credits. After the appointment, the interviewer took notes of rapport between interviewer and interviewee, non‐verbal aspects, disturbances and additional information about the appointment, and digitalised the questionnaire with additional checks for errors.

### Sample

We guesstimated the sample size^4^ based on the suggestions of Malterud et al. (2016), considering breadth of the study aim (broad), sample specificity (low), use of established theory (no), quality of dialogue (unknown) and analysis strategy (cross‐case). The determined number of participants was a priori set to 25, which is within the typical sample size range for interview studies (Kvale, 1996), and later extended to 28 due to the number of people interested in participating.

Eligible participants had to be of legal age, speak German at C1 level, have self‐reported experience with menstrual distress (a formal diagnosis was not required^5^) and have been in a romantic relationship for at least 6 months so that menstrual distress could become noticeable within the relationship. Furthermore, only participants who did not use hormonal contraception for menstrual cycle regulation were recruited, as it can be difficult to distinguish menstrual symptoms from side effects of contraceptives (Sabatini et al., 2011). Finally, participants had to be able to attend in person to ensure consistency of setting and consistent rapport development (cf., Miller, 2017).

### Data analysis

#### Analytical approach

The interviews were transcribed manually and verbatim following guidelines by Dresing and Pehl (2018). Interview transcripts were analysed using qualitative content analysis by Mayring (2015), providing a systematic approach to reduce and structure large data sets and to identify relevant categories within data (Flick, 2014). Inductive coding^6^ was performed in QCAmap (version 1.2.0; Fenzl & Mayring, 2017) by the same researcher who conducted the interviews, supported by a second researcher through multiple intercoder conferences, in which a subset of the material coded by both researchers was discussed until consensus was achieved. Given the abundance of information received from the interviews, we divided the coding process into two steps: (1) data structuring and condensation within the context of the present study and (2) identification of broader patterns across the condensed data which were mainly deducted from reviewing categories and discussions in intercoder conferences. This two‐step approach was chosen to simultaneously allow for an in‐depth analysis of the material based on the research questions (categories, results presented in the Data S1) and a condensed summary of relevant insights contained across categories (broader patterns, presented in the main text).

#### Data structuring and condensation

We inductively developed a coding frame to systematically sort through the data by (1) defining guiding research questions, (2) agreeing on definitions of selection criteria (i.e., which information is relevant for this specific research question), (3) agreeing on a level of abstraction for relevant information (i.e., when assigning relevant information to a ‘category’, how closely does the category name reflect the original text vs. prescind from it) and (4) developing main categories (i.e., grouping identified categories for each research question). See the OSF project for the coding frame.

Our guiding research questions were as follows: (1) How do romantic relationships support coping with menstrual distress? (2) How do romantic relationships strain coping with menstrual distress? (3) How does menstrual distress strengthen romantic relationships? and (4) How does menstrual distress strain romantic relationships? Additional factors were identified that contributed to the relevance of menstrual distress within romantic relationships, such as contextual factors where menstrual distress was perceived relevant (inside and outside romantic relationships) and reasons for talking about menstrual distress in romantic relationships.

Based on the guiding research questions, inductive category formation was achieved by relying on coding units and selection criteria, eventually relabelling categories based on the predefined level of abstraction. Within an iterative process, categories were refined by paraphrasing and summarising information from the interviews and later discussed in multiple intercoder conferences of the two involved researchers to resolve differences. We developed main categories that emerged from grouping identified categories within the interview material for each research question. So‐called *associated categories* represent identified categories that fall under each *main category*.

#### Identification of broader patterns

Using the condensed data, we ultimately identified broader patterns linking menstrual distress and romantic relationships through further intercoder conferences, where the broader patterns were refined and feedback from local health psychology researchers was incorporated after sharing results from both analysis steps (i.e., data structuring and condensation through category and main category formation as well as identification of broader patterns). The feedback refined category definitions, supported partial re‐organisation of categories and coherence of the categorisation.

#### Data quality

Procedural validity was established in this study through interview training, using an interview guide, and standardising the transcription process. Moreover, communicative validity establishes trustworthiness through social discourse (Flick, 2014). In this study, social discourse accompanied multiple stages of study, including discussion of interview questions and study design with experts in qualitative research, discussion of preliminary categories in intercoder conferences and discussion of the findings with further health psychology researchers. The intersubjective agreement established in social discourse also contributes to reliability and objectivity (Kvale, 1996). Furthermore, both Kvale (1996) and Flick (2014) underline the importance of establishing credibility through contextualisation, which we considered by emphasising the study context.

## RESULTS

### Sample

The final sample consisted of 27 participants (age: *M* = 27.68, *SD* = 9.06) because one participant had to be excluded due to the use of hormonal contraception. Participant characteristics are summarised in Table 1. In brief, relationship duration ranged from 6 months to over 18 years, with most relationships lasting between 0.5 and 5 years. Most participants were in heterosexual (*n* = 23) and monogamous (*n* = 17) relationships; 11 cohabited with their partner, and 1 was in a long‐distance relationship. Relationship satisfaction (range: 4–7; *M* = 6.19, *SD* = 0.79) and happiness (range: 4–7; *M* = 6.30, *SD* = 0.81) were high. The total score of the Menstrual Distress Questionnaire, an indicator of global menstruation‐related distress, yielded a mean of *M* = 27.39 (range: 5–50, *SD* = 11.53), which was above the cut‐off value for clinically relevant distress of 20. Thus, on average, participants reported symptoms and symptom frequency that are clinically relevant, and 75% of participants exceeded the cut‐off value for clinically relevant menstrual distress.

**TABLE 1:** Participant characteristics (*n* = 27).

Characteristic		*N* count	*N* %
Demographics			
Age (in years)	≤20	3	11.1
	21–30	20	74.1
	31–40	0	0
	41–50	3	11.1
	>50^b^	1	3.7
Gender	Woman	25	92.6
	Diverse	2	7.4
Occupation^a^	In training/education/studies	18	66.7
	Employed	13	48.1
	Unemployed/seeking work	2	7.4
Sexual orientation^a^	Heterosexual	17	63.0
	Bisexual	7	25.9
	Pansexual	5	18.5
Romantic relationship			
Duration (in years)	0.5–5	17	63.0
	>5–10	9	33.3
	>10–15	0	0
	>15–20	1	3.7
Type^a^	Heterosexual	23	85.2
	Homosexual	2	7.4
	Open	5	18.5
	Committed	6	22.2
	Monogamous	17	63.0
	Queer	3	11.1
	Casual	1	3.7
Shared household	Yes	11	40.7
	No	14	51.9
	Partly	2	7.4
Long distance	Yes	1	3.7
	No	26	96.3
Relationship satisfaction (scale: 1–7)	1–3	0	0
	4–5	4	14.8
	6–7	22	81.5
	Missing	1	3.7
Relationship happiness (scale: 1–7)	1–3	0	0
	4–5	4	14.8
	6–7	23	85.2
Menstrual distress			
Diagnosed conditions^a^	Premenstrual syndrome	1	3.7
	Endometriosis	2	7.4
	Other^c^	6	22.2
	No diagnosis	19	70.4

^a^ Multiple responses were possible.

^b^ Menopausal age is usually between ages of 45–55 years but underlies inter‐individual variability and its onset can be difficult to detect due to menstrual irregularities (Ceylan & Özerdogan, 2015). Thus, 59 years is above average menstruating age, but assuming truthfulness, the shared experiences contribute to the diversity of data.

^c^ Other diagnoses (self‐reported) included cysts, polycystic ovary syndrome, corpus luteum insufficiency, elevated DHEAS levels, postsurgical adhesions, intermenstrual bleeding and irregular menstruation.

### Data structuring and condensation

To facilitate drawing core conclusions, we focus on the broader patterns in the manuscript and only present a shortened form of the data structuring and condensation step (i.e., main categories and associated categories). The latter is described in more detail in Data S1.

See Table 2 for an overview of the main categories. Each cell corresponds to one research question with main categories listed as bullet points. Because many participants reported contextual factors that contributed to the relevance of menstrual distress within romantic relationships, we chose to incorporate them to provide deeper contextual insights (cf., bottom of Table 2) that are also relevant for the identification of broader patterns.

**TABLE 2:** Data structuring and condensation: overview of main categories.

Direction	Support/strengthening	Strain
How do romantic relationships support vs. strain coping with menstrual distress?	Direct support (i.e., active partner behaviour and collective coping) Indirect support (i.e., romantic partners providing space for menstrual distress, contributing to changes in own behaviour of the menstruating person)	Arising negative feelings, worries and fears Arising stressors (i.e., along with interaction patterns, along with intimacy, and along with partners' behaviour)
How does menstrual distress strengthen vs. strain romantic relationships?	Active communication of needs Being authentic and vulnerable by expressing menstrual symptoms Getting new ideas and perspectives throughout the menstrual cycle	Unfavourable influences on well‐being (i.e., the partners' and the own, as well as additional influences along with homosexual relationships) Unfavourable influences during interaction (i.e., restrictions of intimacy, communication patterns and arising conflicts)
Additional contextual factors	Circumstances within the social environment Person‐related factors Menstrual cycle‐related factors Relationship‐related factors Societal factors	

### Broader patterns

The identification of broader patterns aimed to integrate overarching factors of the research questions and across interviews. Importantly, some interviews also mentioned that being in a relationship did not significantly alter their way of coping with menstrual distress (*n* = 4), and others found that partner support only marginally impacted coping with romantic relationships (*n* = 4), for example, by positively influencing individual symptoms but not significantly affecting the overall impact of menstrual distress. Their responses were retained in the material to reflect the broad range of experiences.

#### Space for authenticity, communication and team spirit are key for menstrual support

Participants repeatedly named three factors associated with feeling supported with menstrual distress: first, space for authenticity, that is the space to show menstrual symptoms within and outside of romantic relationships, being allowed to express needs that are present at the moment, receiving adequate reactions, and making room for menstrual topics (during shared time and in discussions). Interviewees described how they took on the journey together, for example, by taking decisions on hormonal contraception together or both reading a book on the menstrual cycle and discussing the insights. Anna (27 years, hetero, TS > 20)^7^ described how having space for these discussions reduced the weight of her menstrual symptoms:

I would say that communication has become more open, actually also in my broader environment …. Even in my workplace—I always work with men who are older … and I'm technically the only woman on the team. In the past, I often also thought I had to say I was sick or something, but by now I also communicate it openly there …. I would say it's more open. I don't have to put on a mask anymore. That removes a bit of the pressure and makes it more bearable. Because it is really exhausting to pretend everything is fine all the time. So that [the relationship] has had a positive influence as it happens. Simply because when it's dealt with openly in one's private environment, the mental threshold for addressing it openly in professional contexts too is lower.

Second, bidirectional communication was crucial for participants, meaning that the partner asks questions related to menstruation proactively but is also open to when the menstruating person feels the need to talk about symptoms, thus allowing for the discussion of solutions and ways of coping with menstrual distress, as Dana (21 years, hetero, TS > 20) pointed out:

I believe that in the beginning, there was also more of a taboo surrounding the topic, I also talked about it less then. But in the meantime, I've tried to also be more open about it and to also communicate when I'm not feeling well. That's why I think it is also a big factor—the relationship too—that I can talk about it with my boyfriend and that he's there for me.

Third, team spirit reflected the feeling of coping collectively and searching for solutions together, ultimately leading to a feeling of shared suffering and collective menstrual cycle management, as Franziska (25 years, committed, TS > 20) described:

I think that was sort of the first time in my life that I felt accepted and that I had the feeling: ‘Alright, we're a team and there are two of us now! We're all in this together’ And that was great! And I think I cope much better because of this mentality. And I do think that the better I feel mentally, the easier and more manageable the symptoms become. And of course, when I'm bleeding and when I'm not depends on my hormones. But how I deal with it is also very important, and I think that without my partner's support, I wouldn't have quite managed.

Importantly, the feeling of shared suffering can be particularly supportive in homosexual and queer relationships. For example, interviewees including Helena (20 years, queer, TS < 20) reported that their relationship offered a basis to fulfil the key aspects of authenticity, communication and team spirit, just because their partner was experiencing the same:

Because I have a partner who also menstruates, it is indeed more comforting. It's that you have a partner who understands, to whom you don't have to explain. And if I say, for example: ‘I'm in pain, I want to lay in bed the whole day …’, my partner just understands, without any need of justification.

#### Supportive factors are a part of healthy relationships

Factors such as having an understanding and interested attitude towards the (menstruating) partner, offering support and showing acceptance of symptoms, needs and boundaries and being able to discuss what preoccupies one's mind were perceived as essential parts of healthy relationships. In this regard, Flora (28 years, hetero, TS < 20) described the interest of her partner in menstrual distress as ‘human interest’. Furthermore, she commented on how menstrual distress (and specifically mood swings as a menstrual symptom) had become part of her personality that her partner had fully accepted:

So my mood, … sometimes triggered by my cycle, definitely influences a lot …, evenings when I'm quite certain that … because of my cycle I'm incredibly irritated and just say no to everything, or feel angry, or annoyed, or bored …. It is part of my personality for him and therefore he likes it as part of my personality. I'm really honest and also speak up if something is annoying or bothering me and I don't hold back.

This perception was shared by other participants who reflected on the relevance to ‘accept a human as a whole; with all specific needs and unique features without trying to change someone’ (Anna, 27 years, hetero, TS > 20) as well as on supporting each other, even when one person needs more support from time to time.

#### Straining factors are intertwined between romantic relationships and menstrual distress

Regarding straining factors, we identified the pattern that disentangling whether strain emerged from menstrual distress or romantic relationships proved to be difficult, both for participants and for coders. For example, emotional irritability was experienced as causing menstrual distress, but several interviewees mentioned that irritability primarily became visible through interaction with their partners. Viktoria (30 years, hetero, TS > 20) said:

in reality, when I am alone, often I don't really notice how irritated I am. And then all he needs to do is stick his head round the door, and suddenly it all comes up at once. As soon as there is another person, I really notice it. Only then do I actually realize how irritated I am. It is just as if I don't have a projection surface when I am alone.

This interplay might be especially critical if both partners are menstruating and struggle with menstrual distress. Helena (20 years, queer, TS < 20) shared:

Sometimes it's just tiring when both of you are on your period at the same time too. It's just like … you kind of simultaneously bring each other down a bit. … I sometimes find it pleasant to simply wallow with another person and to do nothing. But yes, if you are looking for somebody who constantly cheers you up when you're on your period, it's maybe not the best fit.

Importantly, menstrual distress affected both the menstruating individual and their partner, primarily through reduced well‐being and impaired relationship functioning. Interviewees shared that their partners experienced negative feelings, for example, when witnessing menstrual pain and associated struggles with self‐blame, low self‐esteem and body dissatisfaction. Menstrual distress further strained romantic relationships by increasing conflict and misunderstandings, while menstruation‐related limitations reduced shared quality time through cancelled plans and greater partner dependency.

#### Both active and passive partner behaviour can be straining

Active refusal of support such as the partner denying needs of physical closeness or cravings, expressing annoyance or disappointment when menstrual distress occurs or feeling pressured by the partner to engage in sexual intercourse was experienced as straining. For example, Felicitas (30 years, hetero, TS > 20) shared that her partner responded with frustration when she shared that she was on her period:

It used to be really difficult overall—it just was something that couldn't be talked about. Or only with difficulty. And then there'd be this wave of disappointment or frustration, like, ‘Oh no, is it already that time again?’

Also expected yet omitted behaviour was relevant for experienced strain, such as feeling alone with symptoms because the partner is not able to support or due to ignorance, lack of understanding and interest in menstrual distress, contributed to impeded coping with menstrual distress. For example, such an experience became manifest when the partner interpreted menstrual symptoms as ‘temper, as being overly sensitive, or as unreliability’ (Jutta, 43 years, hetero, TS < 20). Passive behaviour and lack of support were experienced by Felicitas (30 years, hetero, TS > 20): ‘It was like this: Whenever I did not feel well, then [my partner] distanced himself. In the sense of: “Ew!”’ Tessa (19 years, queer, TS > 20) also pointed out that the menstrual phase elevated her perception of feeling alone:

So it's definitely difficult when my partner is dealing with her own problems. No matter whether it is stress, fatigue, or being on our period, or any other reason why we feel pain. It is sometimes difficult because when I'm on my period I feel left alone even though that is not the case. But it's hard for both of us.

Overall, straining factors seemed to emerge dynamically and tiny (passive) interaction patterns could intensify perceived strain.

#### Supportive factors are not only experienced as beneficial

A two‐sidedness of support (i.e., including or leading to strain) was prominent in many interviews. For example, instrumental support (e.g., when a partner educated himself on menstrual distress) was experienced as helpful but was also accompanied by a feeling of guilt because the partner sacrificed their time and energy. For example, Kim (27 years, hetero, TS > 20) shared about her partner: ‘He plays a supportive role and yet I feel bad that the other person has to accommodate me’. Similarly, for Mia (27 years, hetero, TS > 20), it is a ‘process of negotiation’ for when it comes to the level of responsibility that her partner takes to support her during her menstrual phase:

My partner, he generally has a rough idea of when it [menstruation] is coming, but I think we still haven't quite figured out the best way to deal with it, and he's not entirely sure how he can help me with it yet …. At the same time, I don't want him to feel responsible for me during that time, but I would still like a certain level of support. And I think it's exactly this process of negotiation.

Moreover, with respect to informational support, some interviewees found it helpful to receive advice, ideas and encouragement for self‐care, whereas others described this behaviour as patronising and perceived it as straining. Julia (28 years, hetero, TS > 20) described this fine line as follows:

I think, it's tricky when my partner thinks I should rest or I should relax or something, and I think ‘No, I don't need that yet, I'm feeling better again’. I just don't like it when someone tells me what to do. Sometimes it's surely helpful, maybe also a good reminder. But—you're not bleeding right now, you should not tell me how to take care of myself.

Some interviewees also described that increased perception of symptoms due to the partner led to a closer connection to the menstruating body in a positive sense, but the increased perception was also straining. Franziska (25 years, committed, TS > 20) shared:

I do think that through the relationship, and through my partner's support, I've learned to express my needs …. And I have become more connected to my body. Well, I notice smaller emotional changes, some pain that I previously would have shrugged off and accepted as ‘That's just a normal part of it’.

Nevertheless, most interviewees appreciated their partners' attempts to support, no matter whether the partner actually alleviated pain. For instance, Sarah (20 years, hetero, TS > 20) said:

I feel like I can talk to my partner about menstrual distress. He listens and tries to help me. At the same time, I feel like he doesn't understand. And that's always a bit frustrating. But at the same time, it already helps that he's there and listens to me. And it already improves things, because you feel like you've got somebody to talk to and who is also kind of trying to understand you.

#### Experiencing support depends on the partner's reaction

More precisely, it seemed that the partner's reaction contributed to whether some aspects of menstrual distress were perceived as supportive or straining for romantic relationships. Opening up about menstrual distress towards the partner and receiving an understanding and empathetic reaction led to feelings of closeness and thus strengthened romantic relationships, while receiving a dismissive reaction led to additional strain and attempts to conceal menstrual symptoms. For example, being vulnerable and authentic when experiencing menstrual distress was also perceived as strengthening romantic relationships, which Felicitas (30 years, hetero, TS > 20) described as follows:

I would actually say that it [the cycle‐related symptoms] somehow made the relationship closer, because these were also things I had to communicate at some point. And then hearing feedback like: ‘Oh, I see—that's what it is …, that's not a problem ….’ For me, it actually made the relationship even closer, because it sort of tore down another wall that I had built for myself. And that I think many women have to build.

Nevertheless, some interviewees also felt uncomfortable to disclose or explain themselves to the partner. Tanja (41 years, hetero, TS < 20) found it difficult to admit to the partner whenever she felt uncomfortable because of menstrual distress because it was closely tied to the partner's reaction:

To communicate that I'm not feeling well or that I feel uncomfortable is like coming out. And that's always difficult. And then also having to say again that things need to be adjusted to me—like, where is a bathroom, or that I need to change …. That I first have to open up, and second, that once I have opened up, it needs to be taken into consideration …. But it's always a question of: Will be accepted or not. And that I'm allowed to be a woman—truly, that I am fully accepted in my womanhood—and that is, of course, associated with a certain uncertainty, but at the same time also very beautiful.

Similarly, interviewees experienced the pressure to hide or justify symptoms towards a partner as straining. Marie (24 years, hetero, TS > 20) shared that she sometimes felt like she had to apologise for her period: ‘As a feminist, I sometimes feel quite ashamed by the fact that I apologize for being on my period, particularly regarding sex’. Anna (27 years, hetero, TS > 20) admitted that it was very difficult for her to communicate openly with her partner about menstrual symptoms and needs: ‘I felt uncomfortable because I thought: He might not take it seriously. Or he'll think I'm too sensitive, something like that. But it [talking about it] helps in the long run’.

This latter quote highlights the relevance of the partner's reaction. As most interviewees reported that authentic communication of menstrual distress supported coping with menstrual distress and prevented strain emerging from attempts at concealment, the partner's reaction seemed to become essential for future authentic communication.

#### Contextual factors matter for the interplay of romantic relationships and menstrual distress

Interviewees described how additional contextual factors contributed to the relevance of menstrual distress within romantic relationships. These contextual factors may be rooted within the person (e.g., communication skills, worries, interest in menstruation‐related topics, attitude towards body and menstrual cycle) and their menstrual cycle (e.g., previous experiences, awareness of patterns and predictability, symptoms and level of distress and medication for better symptom management). Even more frequently, the social environment seemed crucial (e.g., opportunity for exchange, shared experiences and support from family members and friends), and relationship‐related factors (duration, shared housing, conflict levels, sense of security and partner's personality) were addressed. For example, lack of exchange opportunities (i.e., sharing menstruation‐related experiences or pieces of advice with menstruating friends or family members), lack of support within the social environment (i.e., medical assistance, friends and family members) seem to increase the relevance of the partner's role for support, while having close friends as important contact points may decrease it. Specifically, Zoe (27 years, hetero, TS < 20) versus Emma (26 years, hetero, TS > 20), described it as follows, respectively:

Because I already have the feeling that nobody cares about it in the medical field. And if my partner was not interested either, I would feel even more alone. That's when it's good to have someone.

Yes, I have another very, very close friend, who is, along with my boyfriend, my second key support person. And I would say that with her, I share even more than with him.

Similarly, how participants generally dealt with symptoms as a personal characteristic seems to affect the importance of menstrual distress within romantic relationships, as Anna (27 years, hetero, TS > 20) noted:

Well, I'm just … wired differently, actually. I'm the type of person who just pushes through somehow. Until it really isn't possible anymore. And because of that, I just played it down for a long time. And at that point I told myself: This isn't that serious or anything. Until it really wasn't possible anymore. And only when my social environment kept telling me: Go see a doctor! Then I finally sought for help.

These examples illustrate how contextual factors affect the interplay of menstrual distress and romantic relationships, thereby putting more or less weight on the partner's role in symptom management or explaining why the topic of menstrual distress is more or less dominant in romantic relationships.

#### Societal factors are reflected in individual romantic relationships

Going even further, societal factors appear to be highly relevant to how menstrual distress is perceived in the context of a romantic relationship. Importantly, societal factors were reflected in straining (i.e., adoption of adverse societal norms in romantic relationships) as well as in supportive (i.e., romantic relationship experiences as contrast to adverse societal norms) aspects of romantic relationships.

Some interviewees mentioned how they lacked normalisation and acknowledgement of their experiences by society. For example, Zoe (27 years, hetero, TS < 20) felt like this with irregular menstrual cycles:

In my opinion, it mingles somewhat with my identity or with my femininity. … I sometimes feel like an alien or a broken robot. Menstruation is something so clear that is always talked about in such a clear way. And then I feel like I'm sort of outside this norm and not normal, or not represented. That weighs down on me.

Additionally, Felicitas (30 years, hetero, TS > 20) felt that not causing inconvenience due to menstrual distress and hiding menstrual symptoms was the social norm:

Because I was raised to be open, it but still this societal thing of: You're on your period, then you are not allowed to talk about it. … I mean, fifty percent of the population has it [menstruates]. But still such a taboo topic somehow. … because it is not a taboo topic in my relationship, but rather we simply talk about it and deal with it openly and it is not a case of [him saying]: ‘I'll just disappear’ or ‘I can't cope with this right now’ or something, it has made the relationship even closer to me.

This quote illustrates that the contrast of romantic relationships compared with societal influences played a critical role for several participants in a positive sense. That is, destigmatisation and normalisation were rather perceived in romantic relationships but not at a societal level. Normalisation of menstruation‐related aspects in romantic relationships was mentioned in everyday life (e.g., the partner having menstrual products in their apartment's bathroom, providing disposal for menstrual products in the bathroom, considering menstrual phases for planning activities), in conversation with their partners and in being aware of the implications of menstruation.

As an example of normalisation of menstruation in everyday life, Felicitas (30 years, hetero, TS > 20) shared how she appreciated how her partner helped her buy menstrual products: ‘When I say that my menstrual products are almost running out, he just goes to the store without me having to ask for it.’

Regarding conversations, Viktoria (30 years, hetero, TS > 20), who is in a relationship with a male nurse, shared that her partner has a different approach to talking about ‘that stuff’ compared with other men because of his experiences at work. Regarding intimacy levels, Felicitas (30 years, hetero, TS > 20) mentioned her partner's acceptance of not being intimate when she feels uncomfortable:

He's also okay with it when it comes to sexual aspects. For him, it's a natural body function. ‘If you don't feel comfortable with being [intimate], that's okay for me.’ It does not bother him, it's just blood for him.

Overall, it thus seems to largely depend on *how* societal factors are reflected in romantic relationships—either as a positive contrast in terms of destigmatisation and normalisation or as a negative transmission.

## DISCUSSION

Through analysing qualitative interview data from 27 participants who regularly experienced menstrual distress and who had been in a romantic relationship for at least 6 months, we identified factors beyond social support that influenced coping with menstrual distress within romantic relationships and illustrated the interplay with eight broader patterns. Identified factors were not only rooted in the menstruating individual but also in the romantic partner and relationship dynamics as well as in the social and broader cultural context, highlighting that a biopsychosocial perspective is highly relevant for dealing with menstrual distress in romantic relationships.

Romantic partners can facilitate coping with menstrual distress. In line with the literature on coping with pain, chronic conditions more generally and endometriosis specifically, different forms of social support (e.g., practical, emotional, informational and instrumental) were relevant (e.g., Che et al., 2018; Culley et al., 2017; Denny, 2004; Gong et al., 2024). Beyond that, our study pointed to mechanisms that may explain why these factors are relevant in the specific interplay of romantic relationships and menstrual distress. That is, supportive partner behaviour and the partner's eagerness to help often led to a feeling of shared suffering and a sense of team spirit when managing menstrual distress and its impairment, which itself also contributed to supported coping with menstrual distress. This aligns with a recent study showing that perceiving pain as a shared experience was a crucial element in coping with chronic pain within romantic relationships (Carter et al., 2023). By including queer relationships, including relationships where both partners menstruate, the present study highlights that joint understanding of menstrual distress is indeed a resource that supports coping (Ussher & Perz, 2013).

Open communication and authentic expression of menstrual distress were further relevant mechanisms. Menstrual distress affects intimacy, activities, well‐being and interactions, regardless of whether menstruating people disclose their menstrual distress to their partners or not. Open communication seems crucial in navigating through the challenges of menstrual distress together and in avoiding potential misinterpretation of adjustments and changes in daily life due to menstrual distress. Maintaining open communication, however, is closely connected to the partner's reaction. By contributing to the acknowledgement of menstrual symptoms, romantic partners might be able to buffer some impact of societal stigmatisation, trivialisation of menstrual symptoms and the social norm of concealing menstruation. Thus, romantic partners can support menstruating people both directly and indirectly, parallel to the main effect and buffering effect of social support, as demonstrated for chronic conditions (Gong et al., 2024).

At the same time, however, both active (e.g., expression of annoyance or rejection of support) and passive partner behaviours (e.g., freezing due to the feeling of helplessness) may contribute to strained coping with menstrual distress. Passive partner behaviour may result from a feeling of helplessness. Some interviewees in this study described the latter for their partner, noting that when it was not possible to explain or educate their partner due to acute symptoms, the resulting overwhelm and sense of helplessness on the partner's side impeded coping. This was particularly pronounced when participants felt a sense of responsibility for caring for their non‐menstruating partner and regulating their emotions. Other studies have also mentioned the feelings of helplessness for non‐menstruating romantic partners when facing menstrual distress (Clark, 2012; Culley et al., 2017; Peranovic & Bentley, 2017). Experiencing helplessness may reflect a lack of awareness or understanding of menstrual changes and effective ways to support menstruating people, which is potentially rooted in the persistent social norms surrounding menstruation, including its concealment and associated stigma. These social norms likely hinder opportunities for education about menstruation from an early age, perpetuating a cycle of ignorance. The perceived responsibility of menstruating people to inform non‐menstruating romantic partners about menstrual distress and adequate support can feel straining, especially when they experience acute menstrual distress.

Importantly, perceived strain in coping with menstrual distress partially also emerged from anticipated or provided support, namely, when fear to burden the partner or feelings of guilt emerged. This finding aligns with research on chronic pain conditions, which highlighted the potential downsides of social support. For example, studies have shown that social support can exacerbate pain intensity, reinforce pain behaviours and foster dependence on the partner (e.g., Gong et al., 2024; Turk et al., 1992). This two‐sidedness of support emphasises the importance of communication to avoid negative outcomes of well‐intended behaviour. Zee and Bolger (2019) argue that ‘invisible’ support—support that is not perceived as help by the recipient—may be particularly beneficial, as it reduces the risk of these negative outcomes.

The relationship between romantic relationships and menstrual distress is bidirectional; coping with menstrual distress may also affect experiences within romantic relationships. Especially for the straining experiences, the dynamics could hardly be entangled, highlighting that menstrual distress needs to be viewed from a biopsychosocial perspective. Because romantic relationship are inherently intimate, romantic partners are vital in shaping coping with menstrual distress, regardless of whether they actively engage in the topic or not. For example, by ignoring or shying away from the topic, romantic partners also impede coping with menstrual distress, perpetuating the trivialisation of menstrual symptoms. Furthermore, menstrual distress may also affect relationship dynamics in a positive way, for example when a validating partner response leads to the disclosure of menstrual distress and thereby to a sense of emotional closeness, ultimately strengthening the romantic relationship. This aligns with the intimacy model (Cano & d. C. Williams, 2010), which suggests that the expression of pain and distress serves as a mechanism to foster intimacy within relationships. Considering that menstrual distress might be revealed over time anyhow, the partner's response to the disclosure of menstrual distress inevitably also influences the quality of relationships.

### Limitations and directions for future research

The sample was characterised by an overrepresentation of clinically relevant menstrual distress (cf., Vannuccini et al., 2021), the ability to talk comfortably about menstrual distress and high relationship satisfaction. This may have resulted from a self‐selection bias due to increased perceived need for research in this area in individuals who are severely affected or that individuals with low levels of distress felt that they could not meaningfully contribute. A lack of participants with low relationship satisfaction seems common in research (Schick et al., 2022; Turk et al., 1992) because it is often tied to strained partner interactions and may therefore negatively influence communication (Newton‐John, 2013). Because dyadic coping may differ between satisfied and dissatisfied couples (Bodenmann & Perrez, 1991), it is important to note that findings from this study mainly reflect experiences of participants with higher levels of relationship satisfaction. Similarly, the study likely mostly draws on participants who are comfortable discussing menstruation, which may have led to overstating communication as a coping resource. Notably, 22 of 27 participants reported frequently discussing menstrual topics with their partner, contrasting with prior research indicating that such ‘period talk’ with non‐menstruating individuals is often associated with discomfort (Brantelid et al., 2014; Kissling, 1996; Li et al., 2020; Peranovic & Bentley, 2017). This openness may be associated with the relatively high educational status of the sample, which may also reflect greater menstrual health literacy.

Second, our definition of menstrual distress (Vannuccini et al., 2021) primarily focused on symptoms associated with active menstruation, which might have unintentionally excluded specific menstrual disorders such as amenorrhea, that is, the absence of menstruation, another severe but trivialised menstrual disorder (Verhoef et al., 2021). Future research should consider broadening the definition of menstrual distress to include the challenges associated with the absence of menstruation within romantic relationships (e.g., unmet wish to have children or associated future‐related worries), allowing for a more comprehensive view on the interplay of menstrual distress and romantic relationships.

The study also points to several directions for future research, including research exploring the complexities of menstruator—partner interplay to identify the circumstances under which a specific partner behaviour is experienced as supportive or straining. This experience may depend on characteristics of the romantic relationship (e.g., being well‐rehearsed as a team vs. fresh relationship), the menstruating person (e.g., generally accepting a certain feeling of dependency or not; general need for attention and care), the current cycle phase and related symptoms (e.g., higher distress levels during menstruation compared with the follicular phase), day‐specific experiences (e.g., stress at work and conflict with a friend) and many other aspects and may thus be best examined using intensive longitudinal designs.

Moreover, our study hinted at the type of relationship being crucial, such as greater perceived understanding but also downward spirals if both partners experience menstrual distress. Future research should specifically focus on more diverse relationship types and directly study such potential dynamics to contribute to the understanding of contextual factors. Finally, cohabitation was not explicitly addressed in the interview guide. Although the participants rarely mentioning cohabitation as a factor could point towards it playing a minor role, future research should still explicitly explore potential positive (e.g., available practical support) and negative (e.g., lack of space to retreat or avoid conflict) influences to shed further light on this issue.

### Practical implications

Many straining factors were rooted in a lack of awareness of knowledge; thus, initiatives are needed to raise awareness about the role of romantic partners in coping with menstrual distress in line with a biopsychosocial perspective. Most importantly, these should emphasise that romantic relationships affect menstrual distress, regardless of whether couples engage in the topic or not. To avoid putting further strain on menstruating individuals, these campaigns should (also) address non‐menstruating partners and provide practical suggestions for social support to help overcome hesitation and foster more proactive responses. Moreover, our findings suggest that relationship‐related factors may play an important role for menstrual distress. Therefore, relationship‐strengthening interventions that target, for example, communication, conflict management and supportive behaviours, could be an important element for dealing with menstrual distress. Such interventions may not only foster more effective support during menstruation but may also contribute to improved relationship functioning and, in turn, broader health and well‐being among romantic partners. Finally, one may speculate that recent socio‐political changes around menstruation, such as the introduction of menstrual leave policies in several countries including Spain and Sambia, or athletes breaking taboos around menstruation during national and international sporting events such as the 2026 Winter Olympics may support more open conversations about menstruation in different areas of life, including romantic relationships and healthcare context.

This study also underlines the need to improve current programmes for menstrual distress management, which primarily focus on biomedical aspects of menstrual distress and largely neglect the role of social or partner support (e.g., Becker et al., 2022; Kalaitzopoulos et al., 2021). Because menstrual distress can also impact romantic partners (e.g., their well‐being and commitments; cf., Culley et al., 2017; Schick et al., 2022), integrating a couple‐based perspective into guidelines for menstrual distress and recognising the potential of romantic partners in supporting medical treatments as a non‐pharmacological strategy (cf., Che et al., 2018; Gong et al., 2024) seems prudent. In addition, as contextual and societal factors were found to play a significant role, it is important to consider structural factors beyond the interpersonal level. Improved access to menstrual health care and appropriate treatment, as well as training for healthcare professionals to foster safe disclosure, empathetic communication and understanding attitudes, are equally important.

## CONCLUSION

Due to the emotionally and physically intimate nature of romantic relationships, partners influence experiences of menstrual distress, whether actively engaging with the topic or not. Supportive relationships fostering authenticity, communication and teamwork enhance coping, whereas negative partner responses can perpetuate negative stereotypes, stigma and symptom trivialisation. Adopting a biopsychosocial approach that acknowledges the interplay of menstrual distress and romantic relationships for both the impact of menstrual distress on the individual and for coping with menstrual distress as a couple is crucial to promote support and well‐being. Romantic partners can play a crucial role in shaping coping mechanisms for menstrual distress. Consequently, integrating partners into menstrual distress management is vital to help couples navigate the challenges of menstrual distress together, fostering stable, distress‐resistant romantic relationships and enhancing the effectiveness of menstrual distress management.

## CONFLICT OF INTEREST STATEMENT

The authors declare no competing interests.

## ETHICS STATEMENT

Ethical approval for the involvement of human subjects in this study was obtained from the local University's ethics committee.

## Supporting information


**Data S1.** Supporting Information.

## Data Availability

Data collection materials (i.e., interview manual, advertising material and coding scheme; all in German language) are openly available on the Open Science Framework (OSF, https://osf.io/vu5dg/). The data that support the findings of this study are available from the corresponding author upon reasonable request.

## ENDNOTES

^1^
Throughout this study, we refer to menstruating people instead of women to acknowledge that not all people who menstruate identify with the female gender and that not all women menstruate (cf. Wilbur et al., 2019). When referring to previous literature, we use the term mentioned in the studies.

^2^
Selection of relationship types was based on an overview provided by ‘Liebesleben’, an initiative of the German Federal Centre for Health Education. For an explication of relationship types (see https://www.liebesleben.de/fuer-alle/sexualitaet/beziehungsformen/).

^3^
This participant was a researcher employed in the same larger group that conducted this study, but employed in a different project. They expressed interest to participate in the study independent of their job. The interview procedure followed the same standards as these with other participants.

^4^
The gold standard for determining sample size in qualitative research is data saturation, which refers to ‘the point in data collection and analysis when new information produces little or no change to the codebook’ (Guest et al., 2006, p. 65). However, data saturation is difficult to determine (cf., critiques on the lack of adequacy tests to check whether data saturation has been achieved; Guest et al., 2006). Due to the lack of clear standards for determining data saturation in the field, data saturation was not considered in the interview process for determining the sample size.

^5^
Both clinical and non‐clinical menstrual symptoms can significantly impair daily life. In fact, menstrual symptoms can have a negative impact on physiological, mental and emotional well‐being (Matteson et al., 2013) as well as affect quality of life (Sarita et al., 2024) and are thus relevant for our study, independent of diagnosed conditions.

^6^
According to Mayring (2015), both inductive and deductive category formation are guided by the research question and the underlying theoretical framework. In inductive category formation, however, the categories are developed from the material during the coding process rather than being defined a priori. This process is guided by predefined selection criteria and a specified level of abstraction, which determine the aspects of the material considered relevant for category formation. In deductive coding, by contrast, the categories are derived from the research question and theoretical framework and established prior to coding. They are defined a priori and specified through coding rules and examples. In the present study, we followed Mayring's inductive approach, developing the categories systematically from the material while adhering to predefined selection criteria and an established level of abstraction.

^7^
For the purpose of reading facilitation, we used fictive names of participants to guarantee anonymisation. For contextualisation, brackets contain information of interviewees' age, type of relationship, and whether the MEDI‐Q Total Score (TS) exceeds the cut‐off value for clinically relevant menstrual distress.
