# Supplementary material for: Between love and pain: A qualitative analysis of menstrual distress and romantic relationships
Source: Appl Psychol Health Well Being. 2026 Sep 23;18(5):e70219. doi: 10.1111/aphw.70219 (PMC13601718; doi:10.1111/aphw.70219)
Supplement: Supplementary file 1 [file APHW-18-0-s001.docx]

# Supplementary material

# BETWEEN LOVE AND PAIN: A QUALITATIVE ANALYSIS OF MENSTRUAL DISTRESS AND ROMANTIC RELATIONSHIPS

# Data Structuring and Condensation

Within this appendix, we describe the identified main categories of the data structuring and condensation analysis step in more detail for each analysis-guiding research question. Thereby, we also give examples of associated categories for each main category to better illustrate the structuring and condensation process.

## How do Romantic Relationships Support Coping with Menstrual Distress?

Four main categories were identified regarding how romantic relationships supported coping with menstrual distress. Briefly, romantic relationships either contributed to coping with menstrual distress through direct support (i.e., active partner behaviour and collective coping), or through indirect support (i.e., romantic partners providing space for menstrual distress, contributing to changes in own behaviour of the menstruating person).

With respect to direct support, active partner behaviour on the one hand mirrored that most interviewees found it helpful when their partner provided informational support (e.g., menstruation related knowledge or ideas for self-care), emotional support (e.g., listening, encouraging words, expressing interest in menstrual distress), and instrumental support (e.g., buying medication, making tea, preparing hot water bottles). Moreover, proactive help from the partner, taking over household chores and taking responsibility (e.g., organising doctor’s appointments) was perceived beneficial, too. Collective coping, on the other hand, reflected that many interviewees experienced shared preoccupation with the menstrual cycle as supportive (e.g., exploring contraceptive methods together, discussing menstrual issues). Also, the adjustment of joint activities to menstrual distress symptoms was perceived supportive. Importantly, more than the half of interviewees experienced the feeling of shared suffering as making menstrual distress bearable and the certainty of partners’ support led to more ease.

With respect to indirect support, providing space for menstrual distress, on the one hand, described how partners’ awareness for menstrual distress contributed positively to coping with menstrual distress. Interviewees described how their partner’s compassionate attitude towards menstrual distress, their acknowledgement of menstrual symptoms, and their consideration of the menstrual cycle when planning activities helped them in coping with menstrual distress. Several interviewees perceived normalisation of the menstruation (e.g., buying and disposing menstrual products) and respecting boundaries and needs to be beneficial for coping with menstrual distress. In addition, it was perceived as helpful when partners accepted menstrual distress, because it allowed showing authentic behaviour instead of masking symptoms. Changes in own behaviour, on the other hand, reflected that romantic relationships led to a more compassionate approach to menstruation, the menstruating body, and menstrual symptoms, which contributed to improved coping with menstrual distress. For example, multiple interviewees mentioned that because of their partners, they had increased interest in their menstrual cycles and searched for distress coping strategies. Moreover, several interviewees felt more comfortable talking about menstrual symptoms and menstruation related needs thanks to their partner, also outside of their relationships, and learnt to set and communicate boundaries, leading to improved coping.

Nevertheless, some interviews also mentioned that being in a relationship did not significantly alter their way of coping with menstrual distress (N = 4) and others found that partner support only marginally impacted coping with romantic relationships (N = 4), for example, by positively influencing individual symptoms but not significantly affecting the overall impact of menstrual distress. Thus, in spite of the multifaceted ways in which romantic relationships may support coping with menstrual distress, not everyone perceived the benefits.

## How do Romantic Relationships Strain Coping with Menstrual Distress?

Four main categories were identified regarding how romantic relationships strained coping with menstrual distress. Briefly, arising negative feelings, worries, and fears as well as arising stressors (i.e., along with interaction patterns, along with intimacy, and along with partners’ behaviour) negatively affected coping with menstrual distress.

Regarding negative feelings, worries, and fears, several interviewees described how a lack of understanding, empathy, interest, or appropriate responses of the partner (e.g., patronising behaviour) induced negative feelings that impeded coping with menstrual distress. Others shared that they did not feel comfortable expressing menstrual symptoms in front of their partner, leading to a perceived pressure to hide or justify menstrual symptoms. Regardless of whether symptoms were hidden or not, many reported feeling alone with symptoms (in both heterosexual and homosexual relationships) or dependent on the partner (e.g., on their care-taking behaviour). Relatedly, supportive behaviour was often accompanied by a feeling of guilt about partners’ adjustments. Similarly, many interviewees feared that partners’ acknowledgement would decrease over time and that their symptoms would make a negative impression on their partner, including not living up to expectations (e.g., not being able to participate in activities).

Regarding arising stressors, those arising along with interaction patterns included an enhanced perception of distress symptoms, because, for example, irritability (as a menstrual symptom) became more visible. Differences in needs between partners (e.g., rest versus activities) and having to compromise on needs (e.g., not being able to distance within shared housing) were also experienced as straining. Furthermore, the need to educate the partner about menstrual symptoms was experienced as straining for some interviewees. Arising stressors along with intimacy included pain due to a lack of consideration as well as increased symptoms due to contraceptive choices (e.g., copper chain). A few interviewees perceived pressure for intimacy (e.g., not wanting to be intimate during menstruation) or divergent needs for intimacy that strained the ability to cope with menstrual distress. Meanwhile, lack of intimacy due to the partner not wanting to be intimate during menstruation also raised negative feelings such as physical insecurity and rejection. Finally, arising stressors along with partners’ behaviour

included the partner appearing annoyed, disappointed, or angry about menstrual symptoms, and the impression that symptoms were tabooed or stereotyped by the partner. Lack of partner support was also experienced as straining and often attributed to partners’ shame (e.g., feeling awkward to buy menstrual products for the menstruating person), ignorance (e.g., feeling overwhelmed when symptoms like pain occur), general lack of understanding or active refusal.

## How Does Menstrual Distress Strengthen Romantic Relationships?

No main categories were identified regarding how menstrual distress strengthens romantic relationships due to the highly limited number of factors. Nevertheless, some participants (*N* = 3) shared that menstrual distress strengthened their romantic relationships by having to actively communicate needs, being authentic and vulnerable by expressing

menstrual symptoms, or by getting new ideas and perspectives throughout the menstrual

cycle (e.g., negotiations with partner due to shifting needs throughout the menstrual cycle).

## How Does Menstrual Distress Strain Romantic Relationships?

Six main categories were identified regarding how menstrual distress strained romantic relationships. Specifically, unfavourable influences on well-being (i.e., the partners’ and the own, as well as additional influences along with homosexual relationships) as well as unfavourable influences during interaction (i.e., restrictions of intimacy, communication patterns, and arising conflicts) strained relationships.

For unfavourable influences on well-being, many **i**nterviewees mentioned that their partner experienced negative feelings due to menstrual symptoms. For example, it was

difficult for partners to see the menstruating person in pain during the menstruation and not

being able to intervene or provide relief. A few interviewees shared that their partner experienced worries due to menstrual symptoms (e.g., about pregnancies due to irregular cycles). In addition, the majority of interviewees experienced impaired own well-being due to menstrual distress and thus an indirect strain on their romantic relationships. Specifically, struggles with managing restriction and associated self-blame (e.g., for cancelling plans and imposing changes on the partner), difficulties with decision-making, with self-esteem (e.g., feeling unfeminine due to irregular cycles), and with setting boundaries (e.g., taking things more personally during some menstrual phases) impaired own well-being. In homosexual relationships, interviewees reported additional unfavourable influences of menstrual distress. For example, in case of simultaneous menstruation, interviewees experienced mutual symptom intensification leading to a downward spiral of emotions, (perceived) lack of support, and being unable to provide support to their partner. In case of alternating cycles, a cycle synchronisation was mentioned, leading to the perception of shorter cycles.

For unfavourable influences in interaction, restrictions in intimacy – including physical factors (e.g., pain or lack of libido) as well as mental factors (e.g., feeling unclean during menstruation) and practical factors (e.g., increased effort to prevent stains or short time windows due to intermenstrual bleeding) – were perceived as a form in which menstrual distress strained romantic relationships. Additionally, communication patterns were strained due to changes in mood manifested as menstrual symptoms (e.g., being tired, irritated, impatient, mood swings). Some interviewees experienced that mood manifestation transferred to their partner or was projected onto their partner leading to conflicts. Several interviewees described that menstrual distress negatively impacted time with their partner by experiencing reduced enjoyment due to menstrual symptoms, impairments of shared time due to the unpredictability of the menstrual cycle, and changes in relationship dynamics (e.g., increased dependency on partner, reduced quality time due to social withdrawal). Finally, multiple interviewees shared that menstrual distress also strained romantic relationships through arising conflicts, escalation of conflicts, and increased misunderstandings caused by menstrual symptoms.

## Additional Contextual Factors

Many interviewees described additional factors that contributed to the relevance of menstrual distress within romantic relationships. Specifically, circumstances within the social environment (e.g., opportunity for exchange, shared experiences, support from family members and friends), person-related factors (e.g., communication skills, worries, interest in menstruation-related topics, attitude towards body and menstrual cycle), relationship-related factors (duration, shared housing, conflict levels, sense of security, partner’s personality), menstrual cycle-related factors (e.g., previous experiences, awareness of patterns and predictability, symptoms and level of distress, medication for better symptom management), as well as societal factors (e.g., perceived norms, medical assistance, care structure) influenced the relevance of menstrual distress within romantic relationships.

# Main and associated categories per research question

## Research question 1: How do romantic relationships strain coping with menstrual distress?

| **Main Category** | **Associated Category** | **Quote** | **Translation** |
| --- | --- | --- | --- |
| Arising negative feelings, worries, and fears | Pressure to suppress/hide/justify symptoms (e.g., consideration for others, lacking libido) | "Da hatte ich halt einfach das Gefühl, ich kann mich nicht so fallen lassen mit den Schmerzen, oder kann nichts sagen: Boah, es tut gerade richtig weh, oder es krampft voll oder so." (Julia, 28 Jahre, hetero, TS > 20) | “Then, I just had the feeling that I couldn’t flop down with the pain or couldn’t say, ‘Man, it really hurts right now,” or “I have really strong cramps,’ or something.” (Julia, 28 years, hetero, TS > 20) |
|  | Negative feelings due to lack of understanding/empathy/interest (e.g., frustration) | "Und dass er mir auch zuhört […]. Aber gleichzeitig habe ich, wie gesagt, immer das Gefühl, dass es nicht versteht. Und dass ich das auch nicht so wirklich erklären kann. Und dass ist es immer so ein bisschen frustrierend, […], weil ich mich […] unverstanden fühle." (Sarah, 20 Jahre, hetero, TS > 20) | “And that he also listens to me […]. But at the same time, as I said, I always have the feeling that he doesn’t understand it. And that I can’t really explain it either. And that is always a little frustrating […] because I […] don’t feel understood.” (Sarah, 20 years, hetero, TS > 20) |
|  | Negative feelings due to inappropriate responses (e.g., patronizing behaviour) | "Ich glaube, schwierig ist es dann, wenn man Partner denkt, ich sollte mich ausruhen […]. Das mag ich halt einfach nicht, wenn jemand mir sagt, was ich machen soll. Manchmal ist das bestimmt hilfreich, vielleicht auch eine gute Erinnerung. Aber du blutest gerade nicht, du sollst mir nicht sagen, wie ich nach mir gucken soll." (Julia, 28 Jahre, hetero, TS > 20) | “In my opinion, it’s tricky when my partner thinks I should rest […]. I just don’t like it when someone tells me what to do. Sometimes it’s surely helpful, maybe also a good reminder. But - you’re not bleeding right now, you should not tell me how to look after myself.” (Julia, 28 years, hetero, TS > 20) |
|  | Feeling alone with symptoms (including shame, jealousy) | "Also ich sage jetzt nicht […]: ich habe jetzt […] die Menstruation, oder bevor wir uns treffen, das und das geht nicht, sondern in dem Moment, wenn ich jetzt spüre, okay, ich muss jetzt schon wieder aufs Klo, es ist ein bisschen peinlich, weil ich gehe dann öfter aufs Klo, sage ich es halt einfach, ich komme mir dann urblöd vor [...]" (Tanja, 41 Jahre, hetero, TS < 20) | “Well, I don’t say […], ‘I’m currently […] on my period,’ or say I can’t do this or that before we meet up. Rather in the moment when I feel, ‘okay, I need to go to the toilet again’, it’s a bit embarrassing because then I go to the toilet several times and I just say it. I feel like I look like an idiot.” (Tanja, 41 years, hetero, TS < 20) |
|  | Feeling of not meeting the partner’s expectations (e.g., also during time spent together) | "[…] wenn ich mehr darüber nachdenke, ist es schon dieses Ding, dass ich die andere Person damit einschränke. Also, wenn es nur ich bin, dann liege halt ich nur da, aber weil ich mir das dann eben schon nochmal auch wünsche, […] dass die andere Person halt auch da ist, wenn es mir nicht gut geht, oder zumindest, wenn man halt zufällig gerade Zeit gemeinsam verbringt, dass sie dann nicht weggeht." (Kim, 27 Jahre, hetero, TS > 20) | “[…] if I really think about it, the thing is, I limit the other person with it [my period]. Like, when it’s just me, then I just lie there, but because I sometimes do want […] the other person to be there when I’m not feeling well or at least them not to leave if you happen to be spending time together.” (Kim, 27 years, hetero, TS > 20) |
|  | Feeling ill and dependent due to partner's behaviour | "Ich freue mich schon, dass er sich um mich kümmert, aber gleichzeitig bin ich dann halt so: Ich will mir nicht so vorkommen, als wäre ich eben krank oder so, oder als müsste man sich gerade um mich kümmern" (Antonia, 23 Jahre, hetero, TS > 20) | “I’m glad that he takes care of me but at the same time I’m also like, ‘I don’t want to feel like I’m ill or something or like someone needs to look after me right now.” (Antonia, 23 years, hetero, TS > 20) |
|  | Feeling of uncertainty regarding the specificity of requested support | "Und mir fällt es dann immer schwierig, so herauszufinden, was kann ich ihn jetzt […] bitten zu tun oder nicht zu tun. Also gerade, wenn ich zum Beispiel akut Bauchschmerzen habe oder so, dann habe ich ihn gefragt, ob er kochen kann und so. […] Aber dann fände ich es zu viel, zu bitten, zu fragen: 'Kannst du das jetzt bitte um 12 Uhr machen?' Weil ich möchte dann irgendwie abends noch was anderes machen." (Mia, 27 Jahre, hetero, TS > 20) | „And then I always find it hard to find out what I can and can’t […] ask him to do. Especially when I have acute stomach pain or something for example, then I asked him whether he could cook for me and the like. […] But then I’d find it excessive to request, to ask, ‘Can you please do it now at 12 o’clock?’ Because I kind of want to do something else in the evening.” (Mia, 27 years, hetero, TS > 20) |
|  | Guilt toward the partner (due to their adjustments or one’s own behaviour) | "Also er hat es halt auch nicht verdient, wenn ich dann irgendwie gehässig bin oder so. Dann fühle ich mich schlecht, dann entschuldige ich mich wieder, dann passt es wieder." (Viktoria, 30 Jahre, hetero, TS > 20) | „He also hasn’t deserved it when I’m catty or something. Then I feel bad, then I apologise again, then it happens again.” (Viktoria, 30 years, hetero, TS > 20) |
|  | Worries about cycle-related factors (pregnancy, infertility, contraceptive decisions) | "Also ich hatte diese Riesenangst, dass wenn ich schwanger werde, ungewollt, dass ich das erst nach drei Monaten überhaupt merke." (Zoe, 27 Jahre, hetero, TS < 20) | „So I had this massive fear that if I got pregnant, accidentally, I’d only notice it at all after three months.” (Zoe, 27 years, hetero, TS < 20) |
|  | Fear of lacking symptom acknowledgment or loss of understanding | "dass es irgendwann mal nervig wird für die andere Person. […] Das wird sich halt auch nicht ändern, nehme ich an. Ich glaube , das ist so eine Sorge, die ich auch habe, dass ich halt meine Schmerzen habe, wenn die andere Person irgendwie da ist oder so, oder ich halt dann gewisse Dinge nicht machen kann, also nicht noch andere Leute damit beeinträchtigen möchte, und irgendwie so, ja, dass es dann der anderen Person vielleicht nicht auf die Nerven, aber halt so ein bisschen, dann die Geduld damit zu Ende ist." (Kim, 27 Jahre, hetero, TS > 20) | „that it gets annoying for the other person at some point. […] That just won’t change, I assume. I think that’s a worry that I have, that I am just in pain when the other person’s there or something or that I just then can’t do certain things, I don’t want to impair other people too, and kind of that it maybe doesn’t exactly annoy the other person, but just a little, that their patience runs out.” (Kim, 27 years, hetero, TS > 20) |
|  | Worries of negative impression on partner (e.g., being perceived as weak, needy, overly sensitive) | "[…] weil ich so dachte: Der nimmt es nicht ernst, vielleicht. Oder er denkt, ich bin empfindlich, oder so." (Anna, 27 Jahre, hetero, TS > 20) | „[…] because I thought, ‘He might not take it seriously. Or he’ll think I’m overly sensitive, something like that.’” (Anna, 27 years, hetero, TS > 20) |
|  | Fear of burdening the partner (disgust from blood, disappointment, overwhelm) | "Und ich glaube, man hat immer noch den Gedanken im Kopf, dass es eklig ist und dass ich ihm das deswegen nicht zumuten mag oder so." (Emma, 26 Jahre, hetero, TS > 20) | „And I think you still have the thought in your head that it’s disgusting and that I don’t want to put it on him or anything as a result.” (Emma, 26 years, hetero, TS > 20) |
| Arising stressors along with intimacy | (Fear of) occurrence of or misinterpreting physical pain along with used contraceptives | "Und Verhütung ist ein wichtiges Thema. Ich habe jetzt halt auch die Kupferspirale wieder. Ich habe die Kupferspirale mal eine Zeit lang nicht so gut vertragen und habe sie wieder rausnehmen lassen. Und es war mir dann aber alles zu riskant." (Zoe, 27 Jahre, hetero, TS < 20) | „And contraception is an important topic. I have a copper IUD again now. My body hadn’t coped well with a copper IUD at one point, and I had got it taken back out. But then it was all too big a risk for me.” (Zoe, 27 years, hetero, TS < 20) |
|  | Lack of intimacy raises negative feelings (e.g., rejection, physical insecurity) | "[…] ich habe dann wirklich mal gegoogelt: Okay, wenn der Partner keinen Geschlechtsverkehr haben möchte wegen der Blutung, weil er einfach nicht damit umgehen kann, wie löst man das? […] Aber gleichzeitig kann ich […] psychisch und körperlich nicht so gut damit umgehen, dass es sich anfühlt wie so eine Form von Zurückweisung, weil er und ich irgendwie trotzdem eigentlich gerne so Geschlechtsverkehr hätten." (Marie, 24 Jahre, hetero, TS > 20) | „[…] then I genuinely googled: Okay, if your partner doesn’t want to have sex because of your period, because he just can’t handle it, how do you solve it?[…] But simultaneously I […] mentally and physically can’t cope that well with the fact that it feels like some kind of rejection because he and I would technically like to have sex anyway.” (Marie, 24 years, hetero, TS > 20) |
|  | Perceived pressure for intimacy against personal needs | "Weil ist mir schon passiert, dass ich mich bisschen unter Druck gesetzt gefühlt habe: 'ja, du hast wieder deine zwei Wochen Menstruieren, und wir können wieder nicht Sex haben.' (Franziska , 25 Jahre, feste , TS > 20) | „Because it has already happened to me that I’ve felt a bit under pressure: ‘Yes you’re on your two-week period again and we can’t have sex again.’” (Franziska , 25 years, committed, TS > 20) |
|  | Perceived pressure to think about contraception methods / family planning | "Ich bin mir nicht sicher, ob ich Kinder kriegen kann und wenn, dann wird es sehr schwierig. […] Also manchmal liest man von PCO-Patientinnen, die entweder keine Kinder kriegen können oder halt jahrelange Probleme haben […] Ich habe auch gesagt, das möchte ich eigentlich nicht machen. [...] wenn er sagt: 'Ich möchte unbedingt ein natürliches Kind', dann muss ich halt sagen, ich weiß nicht, ob ich das kann. Also das ist dann schon auch so etwas, wo ich sage, ich muss mich jetzt ein bisschen mehr damit beschäftigen, als wenn ich nicht in der Partnerschaft wäre. Dann könnte es mir egaler sein. Aber so habe ich das Gefühl, ich muss mich ein bisschen mehr damit beschäftigen." (Zoe, 27 Jahre, hetero, TS < 20) | „I’m not sure whether I can have children and if so, it will be very difficult. […] Sometimes you read about PCO patients who either can’t have children or just struggle for years […] I also said that I didn’t actually want to do it. […] if he says, ‘I definitely want a biological child,’ then I just have to say that I don’t know whether I can [have one]. So that is also something that I say I have to think about a bit more than if I weren’t in the relationship. Then it could matter to me less. But as it is, I have the feeling that I have to occupy myself with it a little more.” (Zoe, 27 years, hetero, TS < 20) |
|  | Divergent needs for intimacy | "Also was vor allem schwierig ist, finde ich, ist Sexualität. Dadurch, dass es […] bei ihm so über den Tag sich verändert und bei mir halt über Tage, und das überhaupt nicht matcht, also das ist halt super super schwierig ist, dass da halt irgendwie so intuitiv gleichzeitig ein Bedürfnis entsteht." (Lena, 23 Jahre, queer, TS > 20) | „So what is particularly difficult in my opinion is sexuality. As a result of it […] changing throughout the day for him and over multiple days with me and that not matching at all, well, it is just really, really hard for the need to intuitively arise at the same time somehow.” (Lena, 23 years, queer, TS > 20) |
|  | Uncertainties arising along with a practiced open relationship | "Also in dem Sinne das Thema Eifersucht, oder wenn er die offene Beziehung gerade ausgelebt hat, und ich meine, nur weil man eine offene Beziehung hat, ist Eifersucht nicht einfach pauschal kein Thema mehr, sondern schwingt trotzdem mit. Und wenn ich mich eh schon unsicher fühle oder empfindlicher bin, oder emotional traurig bin aufgrund meiner Zyklusphase, und er es dann ausgelebt hat, dann habe ich ein bisschen schlechter damit umgehen können, und es hat mich mehr aufgewühlt, als es gewesen wäre, wenn ich in einer vollstabilen, komfort- und konfidenten Phase bin, und mich einfach gut fühle." (Emma, 26 Jahre, hetero, TS > 20) | „So in that sense the topic of jealousy or when he was just living out the open relationship, and I mean, just because you have an open relationship it doesn’t mean that jealousy is just not relevant anymore, rather it’s involved regardless. And when I’m already feeling insecure or am more sensitive, or I’m emotionally sad because of my phase in the cycle, and he’s then lived it out, then I couldn’t cope with it as well and it agitated me more than it would have if I’d been in a completely stable, comfortable and confident phase and had just been feeling fine.” (Emma, 26 years, hetero, TS > 20) |
| Arising stressors along with partner's behaviour | Lack of support due to shame, unawareness, ignorance | "[…] wenn ich meine starken Schmerzen hatte, war sie meistens immer sehr schockiert und war so: Erstens überfordert, und wusste nicht, was sie tun soll […] und zweitens auch so 'Oh, ich habe nicht gecheckt, dass es so schlimm ist.' […] Also eben auch so eine Hilflosigkeit habe ich dann auch oft gemerkt." (Kim, 27 Jahre, hetero, TS > 20) | „when I was in severe pain she was usually always very shocked and was like: firstly overwhelmed and didn’t know what to do […] and secondly also like, ‘Oh, I didn’t realise that it was that bad.’ […] So I often just noticed a certain helplessness then too.” (Kim, 27 years, hetero, TS > 20) |
|  | Lack of support due to refusal or failure in meeting needs | "Also es war, wenn ich mich nicht wohl gefühlt habe, dann wurde weiter Abstand von mir genommen. Im Sinne von: Ah! (angeekelt). […] Es hat halt einfach nicht gepasst, aber es war gefühlt mehr ein: 'Okay, du bist grumpy, dir geht es nicht gut, dann bin ich mal weg, weil das halte ich jetzt nicht aus.' Es war mehr ein Alleingelassen werden [...]" (Felicitas, 30 Jahre, hetero, TS > 20) | „So it was like this: Whenever I did not feel good, then [my partner] distanced himself. In the sense of: ‘Ew!’ […] It just didn’t fit, but I feel like it was more of a case of ‘okay, you’re grumpy and you’re not feeling well, then I’ll disappear because I can’t cope with this right now.’ It was more of a case of being left alone […]” (Felicitas, 30 years, hetero, TS > 20) |
|  | Partner appears annoyed/disappointed/angry about symptoms | "[…] es war auch irgendwie nicht ansprechbar. Oder schwer ansprechbar. Und dann kam oft irgendwie eine Welle von Enttäuschung oder Ärger mir: 'Ah, jetzt ist es schon wieder so weit?'" (Felicitas, 30 Jahre, hetero, TS > 20) | „[…] it just was something that couldn’t be talked about. Or only with difficulty. And then there’d be this wave of disappointment or frustration, like, ‘Oh no, is it already that time again?’” (Felicitas, 30 years, hetero, TS > 20) |
|  | Symptoms feel tabooed or stereotyped by the partner | "Deswegen finde ich es auch immer schwierig, so, […] ich will mir das von meinem Partner nicht vorwerfen lassen, dass ich deswegen gerade so reagiere" (Jasmin, 23 Jahre, hetero, TS > 20) | „That’s also why I always find it difficult, like, I don’t want my partner to accuse me of reacting like this because of it.” (Jasmin, 23 years, hetero, TS > 20) |
|  | Lack of understanding for the straining situation | "[…] wie sich mein Freund und ich kennengelernt haben, hatte ich eben sehr unregelmäßig meine Periode, das heißt, ich habe sehr selten geblutet. Und das war für Geschlechtsverkehr für meinen Freund irgendwie optimal, weil er sich dann irgendwie viel wohler damit gefühlt hat [...] und das war eigentlich, glaube ich, für ihn als Mann einfach nice, aber für mich eigentlich voll belastend eben und ich hatte das Gefühl, dass da das Verständnis nicht so groß dafür war, warum ich eben eine Blutung haben möchte und warum ich da irgendwie mich bemühen möchte. (Marie, 24 Jahre, hetero, TS > 20) | „when my boyfriend and I met each other I had very irregular periods, that means I menstruated very rarely. And that was sort of optimal for sex for my boyfriend because he kind of felt a lot more comfortable with it […] and I believe it was actually just nice for him as a man, but actually really stressful for me, and I had the feeling that there wasn’t much understanding as to why I wanted to have a period and why I wanted to make an effort towards it.” (Marie, 24 years, hetero, TS > 20) |
| Arising stressors along with interaction patterns | Self-care dependent on or affected by the relationship | "Aber ich glaube, da habe ich auch verschiedene Erfahrungen. Also der, mit dem ich jetzt zusammen bin, ist schon -- der ist sowieso anders. Und nimmt das ganzheitlich auf. Da habe ich schon andere Erfahrungen gemacht. Ich glaube, das war dann auch schwer für mich, mich da selbst anzunehmen. Oder das sein lassen zu können." (Tanja, 41 Jahre, hetero, TS < 20) | „But I think I’ve had different experiences as well. Like, with the one I’m currently with it is - he’s different anyway. And he takes it as a whole. That’s where I have had other experiences. I think that it was hard for me to accept myself then too. Or to be able to let it be.” (Tanja, 41 years, hetero, TS < 20) |
|  | Unmet needs or necessity to compromise needs (e.g., when living together) | "Mein Wunsch ist einfach, dass ich in Ruhe gelassen werde in der Zeit, was natürlich auch nicht ganz geht, wenn man zusammenwohnt, weil man immer irgendwo Berührungspunkte hat." (Viktoria, 30 Jahre, hetero, TS > 20) | „My desire is just to be left in peace at that time which of course also isn’t entirely possible when you’re living together as you always come into contact in some ways.” (Viktoria, 30 years, hetero, TS > 20) |
|  | Differences in needs (including arising potential for conflict) | "generell die Art, von wie energetisch wir beide sind […] an verschiedenen Tagen. Dass er so immer irgendwie so ein Mittelding ist und ich halt eher vor allem auch während meines Eisprungs wirklich sehr, sehr viel machen möchte und ähm, auch sehr, sehr viel und sehr schnell rede, ähm, und irgendwie, da wirklich sich viel bei mir ändert so von Tag zu Tag, Woche zu Woche. Ähm. Und ich da irgendwie manchmal wünsche, dass ich eine Person hätte, die dann sofort aufspringt und die das so mit mir zusammen macht und auch die Art von Energie gerade an den Tag legt, die ich da gerade habe. Ähm. Und das ist manchmal schade." (Lena, 23 Jahre, queer, TS > 20) | „generally in the way of how energetic we both are […] on various days. That he’s always kind of in the middle ground and I just tend to want to really do a great, great deal, especially during ovulation, and uh, also talk a lot and very quickly, uhm, and sort of, as there really are a lot of changes in me like from day to day, week to week. Uh. And then I sometimes kind of wish that I had a person that immediately jumps up then and that does it with me and displays the same kind of energy that I’m currently experiencing too. Uh. And that’s sometimes a shame.” (Lena, 23 years, queer, TS > 20) |
|  | Potential for conflict due to anger about partner's behaviour | "Ich glaube, es ist dann schon oft immer so, dass […] von der anderen Person dann kommt: 'Kann es nicht sein, dass du bald deine Tage bekommst?' Manchmal hilft das auch voll. […] Aber natürlich halt vielleicht, wenn man gereizter ist oder so, ist es halt, jetzt in der Partnerschaft nicht, aber [...] in der Vergangenheit dann schon einfach zu Konflikten gegangen ist. Gerade, wenn es einfach schon aufgeladener war und halt andere Konflikte waren, dann hat sich halt ein bisschen mehr gezeigt in der Zeit, würde ich sagen." (Kim, 27 Jahre, hetero, TS > 20) | I think it’s then always a case of […] the other person then saying, “Is it possible that your period is coming up?” Sometimes that really helps too. […] But of course maybe when you’re more irritated or something it’s just, not in this relationship, but […] it’s just led to conflicts in the past. Precisely when it’s just been more tense and there were other conflicts, then a little more came up in that time, I would say.” (Kim, 27 years, hetero, TS > 20) |
|  | Physical pain due to lack of consideration by the partner | "Insofern wird das dann erst Thema, wenn es zu starke Schmerzen sind oder so, oder wenn man irgendwie, weiß ich nicht, wenn der andere, wenn das Gegenüber Schmerzen auslöst oder beeinflusst, wie beispielsweise, wenn starke Brustschmerzen da sind und eine Umarmung gerade nicht mehr das Richtige ist, eigentlich, weil das irgendwie gerade wehtut, dann muss man das natürlich kommunizieren" (Elisabeth, 45 Jahre, hetero, TS < 20) | „In this respect it only comes up when the pain is too strong or something or when you’re somehow, I don’t know, when the other person is causing or influencing the pain, like if you’re experiencing chest pain and a hug technically isn’t the right thing anymore for example because it kind of hurts in that moment, then you obviously need to communicate that.” (Elisabeth, 45 years, hetero, TS < 20) |
|  | Enhanced symptom perception | "Es ist tatsächlich so, wenn ich alleine bin, merke ich oft gar nicht, dass ich gereizt bin. Und er braucht dann nur den Kopf bei der Tür reinstrecken und es ist alles auf einmal da. Also sobald ich das Gegenüber habe, wird mir das dann richtig bewusst. Dann merke ich eigentlich erst, wie gereizt ich bin. Also quasi so, als hätte ich keine Projektionsfläche, wenn ich alleine bin." (Viktoria, 30 Jahre, hetero, TS > 20) | „In reality, when I’m alone, often I don’t really notice how irritated I am. And then all he needs to do is stick his head round the door and suddenly it all comes up at once. As soon as there’s another person, I really notice it. Only then do I actually realise how irritated I am. It’s just as if I don’t have a projection surface when I’m alone.” (Viktoria, 30 years, hetero, TS > 20) |
|  | Necessity for self-explanation/self-disclosure | "Also, schon zu sagen: 'Gut, jetzt sind diese Tage, oder das, also das einmal zu benennen, dass es mir da irgendwie nicht gut geht, oder dass ich mich nicht wohlfühle, also sich da quasi zu outen, das ist sowieso immer schwer." (Tanja, 41 Jahre, hetero, TS < 20) | „So, to say, ‘It’s that time of the month,’ or to actually express that I’m not feeling well or that I don’t feel comfortable - to basically out oneself in that regard, that is always difficult anyhow.” (Tanja, 41 years, hetero, TS < 20) |
|  | Necessity to educate the partner | "Das ist schon so Vorwissen und dass man Dinge erklären muss in der Partnerschaft, ist schwierig." (Zoe, 27 Jahre, hetero, TS < 20) | „That kind of is prior knowledge and the fact that you have to explain things in a relationship is difficult.” (Zoe, 27 years, hetero, TS < 20) |

## Research question 2: How do romantic relationships support coping with menstrual distress?

| **Main Category** | **Associated Category** | **Quote** | **Translation** |
| --- | --- | --- | --- |
| Behavior of partner (direct support) | Informational support (by providing knowledge, reminders about self-care, feedback and by classifying symptoms) | Weil manchmal finde ich es gut, wenn er mich dann ermutigt und sagt: 'Nein, geh in den Sport, das tut dir gut, und triff dich mit Freund:innen, das tut dir gut.'" (Mia, 27 Jahre, hetero, TS > 20) | „Because sometimes I find it great when he encourages me and says, ‘No, do sports, it does you good, and meet up with friends, it does you good.’” (Mia, 27 years, hetero, TS > 20) |
|  | Taking responsibility | "Entweder, wenn man gerade beispielsweise, wenn man gerade unterwegs ist und es so stark wird: 'Bitte ruf ein Taxi, […] ich kann nicht mehr mit der U-Bahn nach Hause fahren', und das gemanagt zu bekommen und sich nicht mehr selbst darum kümmern zu müssen, also das empfinde ich auch als angenehm." (Elisabeth, 45 Jahre, hetero, TS < 20) | „Either, if for example, if you’re currently out and it gets too strong: ‘Please call a taxi, […] I can’t get home with the subway anymore,’ and to have that dealt with and to not have to handle it yourself anymore, well, I find that pleasant too.” (Elisabeth, 45 years, hetero, TS < 20) |
|  | Emotional support | "[…] wenn ich dann bei ihm bin, holt er mir eine Wärmflasche oder versucht sich um mich zu kümmern und ist dann auch immer da, wenn ich mich zum hundertsten Mal darüber aufrege, dass das jetzt gerade scheiße ist. Er versteht das und versuch mich zu trösten und so. Also die Beziehung wirkt sich somit [...] positiv aus, weil ich eben eine Person habe, der ich ein bisschen mein Herz ausschützen kann, auch wenn es jedes Mal wieder das gleiche ist, was ich erzähle." (Dana, 21 Jahre, hetero, TS > 20) | „[…] when I’m then at his, he’ll fetch me a hot water bottle or try to take care of me and then he’s always there when I complain that it sucks right now for the hundredth time too. He gets that and tries to comfort me and so on. So my relationship has had […] a more positive impact [on menstrual distress] precisely because I have a person whom I can pour out my heart to, even if I’m always saying the same stuff.” (Dana, 21 years, hetero, TS > 20) |
|  | Encouraging to do self-care | "Ich glaube schon, dass viele Frauen dann vielleicht auch eher mehr so Bemerkungen haben: 'Stell dich nicht so an, kann gar nicht so schlimm sein, tu was - mach das oder das' Und das ist halt gar nicht, im Gegenteil. Ich versuche immer, dass ich mich so wenig wie möglich einschränken lasse, und werde da auch eher gebremst, dass es dann heißt: 'Mach das und das, oder tu einfach gar nicht'. Und das empfinde ich schon als sehr wertvoll, tatsächlich." (Anna, 27 Jahre, hetero, TS > 20) | „I do think that lots of women may also experience comments more along the lines of, ‘Don’t make such a fuss, can’t be that bad, do something - do this or that.’ And that’s just not [true] at all, the opposite. I always try to let myself be limited as little as possible and tend to then be slowed down, so people then say, “Do this or that or just don’t do it at all.’ And I do find that very useful actually.” (Anna, 27 years, hetero, TS > 20) |
|  | Taking over household chores and reducing daily stress | "Wie zum Beispiel, wenn ich mein kleines Packerl zu Hause habe, wo ich weiß, da ist wenigstens ein Essen drin, dann ich mich jetzt nicht zum Supermarkt oder so schleppen". (Anna, 27 Jahre, hetero, TS > 20) | „Like for instance, if I have my small package at home that I know there’s some food in at least, then I don’t have to drag myself to the supermarket or anything.” (Anna, 27 years, hetero, TS > 20) |
|  | Instrumental support | "Er hat sich da auch auseinandergesetzt damit und er hat mich eigentlich auch mehr oder weniger zu dem Arzt manövriert, wo ich jetzt gerade bin und der ist eigentlich sehr, sehr gut." (Anna, 27 Jahre, hetero, TS > 20) | „He educated himself on [endometriosis/menstrual distress symptoms] and more or less dragged me to my current doctor. He [the doctor] is actually very, very good.” (Anna, 27 years, hetero, TS > 20) |
|  | Fulfilling needs | "Ich habe das Gefühl, insgesamt tut es [die Partnerschaft] mir gut, weil ich dann auch öfters ein größeres Nähebedürfnis habe und das kann dann quasi gestillt werden" (Madeleine, 29 Jahre, hetero, TS > 20) | „I get the feeling that, as a whole, it [the relationship] does me good because I then often have a greater need for intimacy and that can then pretty much be satisfied.” (Madeleine, 29 years, hetero, TS > 20) |
|  | Offering support | "ich weiß nicht, wie er es macht, es ist einfach eben -- es ist ein Ding von: er möchte mir einfach helfen, er möchte einfach für mich da sein, und auch allein in diesem: 'Kein Problem, erzähl mir, was los ist, lass uns doch heute das auslassen, lass uns doch irgendwie schauen, dass es dir gut geht, was kann ich für dich tun, erzähl!'" (Felicitas, 30 Jahre, hetero, TS > 20) | „I don’t know how he does it, it’s just - it’s a case of him just wanting to help me, him just wanting to be there for me, and even just in this, ‘No problem, tell me what’s up, let’s skip this today after all, let’s make sure that you feel okay, what can I do for you, tell me!’” (Felicitas, 30 years, hetero, TS > 20) |
| Collective coping with menstrual cycle and menstrual distress within romantic relationships (direct support) | Collective coping and preoccupation with menstrual cycle and menstrual distress | "[…] ich würde sagen, er merkt […] recht schnell, wenn ich nicht gut drauf bin, und dann sagt er: 'Hey, komm, wir setzen uns mal hin und reden darüber.' Und dann reden wir halt wirklich auch teilweise lange darüber und versuchen irgendwie eine Lösung zu finden." (Sarah, 20 Jahre, hetero, TS > 20) | „[…] I would say he notices […] relatively quickly when I’m not feeling great and then he says, ‘Hey, come on, let’s sit down and talk about it.’ And then we do talk about it for what can really be quite a long time and try to find a solution somehow.” (Sarah, 20 years, hetero, TS > 20) |
|  | Feeling of shared suffering | "die Partnerschaft kann dann halt wieder dabei helfen, dass man die Beschwerden halt besser --- irgendwie, auch wenn man sie nicht beheben kann oder verschwinden lassen kann, aber dann kuschelt man sich halt auf der Couch irgendwie zusammen, und dann sind sie halt besser erträglich." (Pauline, 21 Jahre, hetero, TS > 20) | „the relationship can also help you better - somehow, even if you can’t get rid of the symptoms or make them disappear, then you can just cuddle up on the sofa and then they’re just more bearable.” (Pauline, 21 years, hetero, TS > 20) |
|  | Flexible menstrual-cycle based shaping of couple time | "Und ja, sowas eher halt, und dann andere Sachen von, wie man auch miteinander redet, würde ich sagen, besonders wenn ich zum Beispiel eher so emotional bin, und dann halt von so, okay, was ich in dem Moment gerade eher brauche, und weil das eben auch so schwankend ist. Manchmal will ich dann komplett rausgehen und irgendwie was machen und voll aktiv sein, und manchmal will ich schon, nein, ich will jetzt einfach drinnen sein und einfach nichts machen. Und dass man sich sozusagen irgendwie dem anpasst." | „And yes, more like that, and then other things like how you talk to each other, I would say, especially when I’m rather emotional for example, and then just about, like, what I need more in that moment, and because that’s also so variable. Sometimes I really want to go out and do something and be totally active, and sometimes I want to, no, I just want to be inside right now and just not do anything. And that you basically kind of adjust to that.” |
|  | Certainty of support 🡪 relief of mental load | "wie gesagt, in den letzten anderthalb Jahren sind eben die Beschwerden für mich jetzt nicht mehr so ausschlaggebend, weil es ist einfach ein Ding von, wenn es mir nicht gut geht, dann ist er da und versteht das. Und fängt mich auf." (Felicitas, 30 Jahre, hetero, TS > 20) | „as I said, in the last one and a half years the symptoms weren’t as big a deal anymore because it’s just a case of if I’m not feeling well, then he’s there for me and understands. And picks me up.” (Felicitas, 30 years, hetero, TS > 20) |
| Change in own behaviour and attitude toward menstrual cycle because of romantic relationship (Indirect Support) | Preoccupation with menstrual cycle, changes throughout menstrual cycle and menstrual distress because of partner | "Also er hat das Thema auch in Gesprächen sehr in den Fokus gerückt, indem er sehr viel nachgefragt hat […] und ich ihm dadurch sehr viel Wissen vermittelt habe, aber mir auch gleichzeitig viel bewusst geworden ist durch diese Gespräche und ich mich dann auch selbst nochmal mehr informiert habe und mehr in das Thema eingelesen habe." (Emma, 26 Jahre, hetero, TS > 20) | „So he also focused on the topic a lot in conversations by asking a lot of questions […] and I passed a ton of knowledge onto him as a result, but at the same time I became aware of a lot through these conversations as well and I then also informed myself some more and read up on the topic more.” (Emma, 26 years, hetero, TS > 20) |
|  | Preoccupation with own needs because of partner | "Dass er sich auf mich einstellen möchte, das spornt mich auch dazu an - dadurch muss ich das auch erstmal wissen. Oder ich möchte mich erstmal damit auseinandersetzen, was will ich jetzt eigentlich gerade und bringt mich da in eine Selbstreflexion, indem ich ihm das auch erstmal mitteilen muss." (Emma, 26 Jahre, hetero, TS > 20) | „The fact that he wants to adapt to me, that also encourages me to do it - as a result I need to know it in the first place. Or I would like to spend some time on identifying what I actually want right now first and that triggers self-reflection as I have to share that with him as well.” (Emma, 26 years, hetero, TS > 20) |
|  | Preoccupation with coping strategies because of partner | "Und ich glaube in dem Sinne, dass ich wirklich einfach mehr darüber rede und versuche auch eine Lösung zu finden. Oder irgendwie mit mir selbst einen möglichst guten Umgang zu finden" (Sarah, 20 Jahre, hetero, TS > 20) | „And I think in the sense that I really just talk about it more and also am trying to find a solution. Or to kind of find the best possible way of handling myself.” Sarah, 20 years, hetero, TS > 20) |
|  | Communication about menstrual symptoms because of partner | "Es ist offener, tatsächlich. Ich würde sagen, die Kommunikation ist offener, auch in meinem Umfeld, tatsächlich […]. Also, auch in meinem Arbeitsumfeld, tatsächlich, ich arbeite halt immer mit Männern zusammen, die halt älter sind, die sind alle 40+, und ich bin eigentlich die einzige Frau im Team. Auch da habe ich mir halt oft gedacht, ich bin halt krank oder so, aber mittlerweile kommunizieren ich es auch da offen. [...] Also, das hat es schon tatsächlich positiv beeinflusst. Einfach weil, wenn im privaten Umfeld offen damit umgegangen wird, dann ist die Hemmschwelle, das im Berufsleben auch offen auszutragen, eine geringere, würde ich sagen." (Anna, 27 Jahre, hetero, TS > 20) | „I would say that communication has become more open, actually also in my broader environment […]. Even in my workplace - I mainly work with men who are older, they are all aged 40+, and I’m technically the only woman on the team. In the past, I often thought I was ill there too or something, but by now I also communicate it openly there. […] So that [the realtionship] has had a positive influence as it happens. Simply because when it’s dealt with openly in one’s private environment, the mental threshold for addressing it openly in professional contexts too is lower, I would say.” (Anna, 27 years, hetero, TS > 20) |
|  | Acting based on needs and setting boundaries because of partner | "Er veranlasst mich dazu, klarer zu kommunizieren, […] besser und klarer für mich einzustehen und abzugrenzen, wenn es mir nicht gut geht, damit es eben nicht zu Konfliktsituationen kommen, weil letztendlich sind das Zustände, wo er, glaube ich, mir auch nicht helfen kann, weil das einfach vorbeigehen muss." (Jutta, 43 Jahre, hetero, TS < 20) | „He causes me to communicate more clearly, […] to stand up for myself better and more clearly and to draw the line when I’m not feeling well so that it doesn’t come to conflict situations because at the end of the day these are circumstances where he, in my opinion, can’t help me either because it just needs to pass.” (Jutta, 43 years, hetero, TS < 20) |
|  | Feeling of acceptance towards body and menstruation because of partner | "Positiv. Positiv, weil ich merke, dass das [Menstruieren] dazugehört und dass das zugehören darf zu mir und der Zyklus muss nicht so sein, dass er weh tut, sondern schön […] also ich habe noch keine Kinder, das hat bei mir noch nicht gepasst, wir reden darüber, dass es noch möglich ist bei mir [...] also dass es gut ist, einen Zyklus zu haben, wenn ich noch gebärfähig bin." (Tanja, 41 Jahre, hetero, TS < 20) | „Positively. Positively because I recognise that it [menstruating] is part of life and this part is allowed for me and that the menstrual cycle doesn’t have to hurt, but rather [is] beautiful […] well, I don’t have any children yet, it just wasn’t right for me yet, we are talking about the fact that it’s still possible for me […] so that it’s good to have a cycle if I can still conceive.” (Tanja, 41 years, hetero, TS < 20) |
|  | Communication of needs because of partner | "Also ich denke schon, dass ich durch die Partnerschaft und durch die Unterstützung von meinem Partner gelernt habe, meine Needs, was ich brauche, zu äußern." (Franziska, 25 Jahre, feste, TS > 20) | „So I do think that I’ve learned to express my needs, what I need, as a result of the relationship and because of my partner’s support.” (Franziska, 25 Jahre, committed, TS > 20) |
|  | Change of perspective because of partner | "Und er kennt mich da schon auswendig, so, was meine Beschwerden sein werden und alles Drum und Dran. Und ich glaube, es ist in dem Sinn wichtig, manchmal halt eben diese Außenperspektive zu manchen Sachen zu bekommen." (Pauline, 21 Jahre, hetero, TS > 20) | „And he already knows me off by heart, like, what my symptoms will be and the whole lot. And I believe that in this sense it’s important to sometimes just get this outside perspective on some things.” (Pauline, 21 years, hetero, TS > 20) |
| Space for menstrual distress (Indirect support) | Partner's awareness | "Ja, vielleicht, dass er manchmal besser auf dem Schirm hat, dass ich bald meine Tage bekomme, und er dann fragt oder so: 'Wie sieht es eigentlich mit deiner Temperatur aus?' Ja, dass er mich dann so bisschen drauf bringt" (Julia, 28 Jahre, hetero, TS > 20) | “Yes, perhaps that he sometimes he’s more aware that my period is coming up and then he asks, ‘What’s you temperature?’ Yes, that he kind of makes me realise it.” (Julia, 28 years, hetero, TS > 20) |
|  | Empathetic attitude of partner (e.g., understanding, acceptance) | "Ich habe das Glück, dass mein Partner sehr verständnisvoll ist, was das betrifft." (Anna, 27 Jahre, hetero, TS > 20) | „I am lucky that my partner is very understanding with regards to that.” (Anna, 27 years, hetero, TS > 20) |
|  | Normalizing menstrual cycle, menstruation, and menstrual distress by partner | "ich habe mich von ihm nie abgelehnt gefühlt […], wegen Tagen oder so. Er hat das immer natürlich behandelt und er sagt halt, dass es nicht ekelhaft ist oder so." (Antonia, 23 Jahre, hetero, TS > 20) | „I’ve never felt rejected by him […] because of my period or something. He’s always handled it in a natural way and says that it’s not gross or anything.” (Antonia, 23 years, hetero, TS > 20) |
|  | Partner's acknowledgement of menstrual symptoms | "Bei mir ist es halt so, dass jetzt auch mein Partner das nicht so abtut oder irgendwie belächelt oder so, sondern er sieht halt, dass es wirklich ein Ding ist und er versteht es auch." (Klara, 24 Jahre, hetero, TS > 20) | „For me it’s a case of my partner not dismissing it or making fun of it or anything either, instead he sees that it’s genuinely an issue and he gets that too.” (Klara, 24 years, hetero, TS > 20) |
|  | Respecting boundaries and needs by partner | "Mein Wunsch ist einfach, dass ich in Ruhe gelassen werde in der Zeit, was natürlich auch nicht ganz geht, wenn man zusammenwohnt, weil man immer irgendwo Berührungspunkte hat. Aber er versucht dann trotzdem, so gut es geht, einfach dem gerecht zu werden. Wenn ich dann sage, ich brauche einfach einen Abend jetzt für mich, dann passt das auch." (Viktoria, 30 Jahre, hetero, TS > 20) | „My desire is just to be left in peace at that time which of course also isn’t entirely possible when you’re living together as you always come into contact in some ways. But he tries to live up to that as much as possible regardless. If I then say that I just need an evening to myself right now, then that‘s also fine.” (Viktoria, 30 years, hetero, TS > 20) |
|  | Feeling of acceptance allows authentic behaviour | "Und ich würde sagen, es ist offener. Ich muss jetzt nicht mehr so gezwungen diese Fassade aufrechterhalten, würde ich sagen. Was tatsächlich auch dem Ganzen so ein bisschen den Druck rausnimmt und es ein Stück weit erträglicher macht. " (Anna, 27 Jahre, hetero, TS > 20) | „I would say I am not obliged to put on a mask anymore. Which actually also removes a little of the pressure from it all and makes it a bit more bearable.” (Anna, 27 years, hetero, TS > 20) |

## Research question 3: How does menstrual distress strengthen romantic relationships?

| **Main Category** | **Associated Category** | **Quote** | **Translation** |
| --- | --- | --- | --- |
| Strengthening relationships by… | Actively communicating needs | "Ich würde tatsächlich sagen, dass es irgendwie die Partnerschaft noch enger gemacht, weil auch das irgendwie Sachen sind, die ich kommunizieren musste an irgendeinem Punkt" (Felicitas, 30 Jahre, hetero, TS > 20) | „I would actually say that it somehow made the relationship even closer because they were sort of things that I had to communicate at some point.” (Felicitas, 30 years, hetero, TS > 20) |
|  | Showing oneself vulnerable | "[…] hat es für mich die Partnerschaft sogar noch enger gemacht, weil es hat wieder irgendwie eine Grenze eingerissen, die ich für mich selbst auch irgendwie geschaffen habe, und die, glaube ich, auch viele Frauen für sich selbst schaffen müssen." (Felicitas, 30 Jahre, hetero, TS > 20) | „It has actually made the relationship even closer because it somehow tore down another wall that I had built for myself and that I think many women have to build.” (Felicitas, 30 years, hetero, TS > 20) |
|  | Receiving new ideas and changing perspectives throughout the menstrual cycle | "Aber ich glaube, dass der Zyklus an sich […] auch was Positives darstellen kann. Dadurch, dass […] durch den Zyklus immer wieder neue Ideen entstehen. Und andere Blickweisen auf die Beziehung möglich sind. Dadurch, dass Hormone sich ändern." (Lena, 23 Jahre, queer, TS > 20) | „But I think the menstrual cycle […] can also represent something positive [for the romantic relationship]. […] because […] of the cycle, new ideas are constantly created and other perspectives on the relationship are possible. Because hormones change.” (Lena, 23 years, queer, TS > 20) |

## Research question 4: How does menstrual distress strain romantic relationships?

| **Main Category** | **Associated Category** | **Quote** | **Translation** |
| --- | --- | --- | --- |
| Unfavourable influences on partner's well-being | Partner experiences negative feelings due to inadequate handling or lack of possibilities to intervene | "Er meinte vor allem, dass es bei ihm [...] ein Gefühl von Angst hervorruft, dass er halt Blut sieht und wenn die Blutung stark ist, dann wirklich halt wie als wäre eine Verletzung da, und er sich irgendwie schwer tut damit, das so ein bisschen auseinanderzuhalten. Ist das jetzt eine Gefahrensituation, also rein instinktiv?" (Marie, 24 Jahre, hetero, TS > 20) | „Above all he said that it […] triggers a feeling of fear in him, seeing blood, and when it’s heavy bleeding, then [it’s] really like there were an injury, and he sort of struggles a little to differentiate it. Is it a dangerous situation, like purely on an instinctual level? Above all he said that it […] triggers a feeling of fear in him, seeing blood, and when it’s heavy bleeding, then [it’s] really like there were an injury, and he sort of struggles a little to differentiate it. Is it a dangerous situation, like purely on an instinctual level?” (Marie, 24 years, hetero, TS > 20) |
|  | Partner experiences negative feelings due to one's behaviour (e.g., need for distance, reduced intimacy) | "also ich wolle dann zum Beispiel nicht kuscheln und so, also ich bin halt nicht so nähebedürftig und ich bin dann halt so: 'Geh zurück. bitte.' Am Anfang hat er immer so gedacht, es hat was mit ihm zu tun oder ich mag ihn nicht mehr so oder solche Dinge." (Antonia, 23 Jahre, hetero, TS > 20) | „so I don’t want to cuddle, for example, or something, so I just don’t have such a need for closeness, and then I’m just like, ‘Get back, please.’ At the beginning, he always thought it had something to do with him or that I don’t like him or something like that.” (Antonia, 23 years, hetero, TS > 20) |
|  | Partner is burdened (e.g., time constraints, emotional outbursts) | "Und zwar nicht unbedingt in dem Moment selbst, wo er sich um mich kümmert, weil es das sichtbar gerne macht, sondern weil es ein Stressfaktor ist, wenn eigentlich doch noch was anderes anstehen würde, aber er sieht, dass es mir nicht gut geht. Und dann sind es plötzlich vier, fünf Sachen mehr, die er an dem Tag machen muss, als geplant. Also 'muss'. Aber es ist einfach. Und das ist dann eher der Strain auf die Beziehung. Ich weiß ja dann, dass ich ihn auch belaste. Gleichzeitig kann ich in dem Moment auch nicht gerade was anderes machen." (Madeleine, 29 Jahre, hetero, TS > 20) | „Not necessarily in the moment, when he is taking care of me, itself, as he clearly enjoys doing that, rather because it’s a source of stress when something else technically still needs doing but he can tell that I’m not feeling well. And then there are suddenly four, five more things that he needs to do that day than planned. Well, ‘needs to’. But it is just [like that]. And that’s then more of a strain on the relationship. I know then that I’m putting a strain on him. At the same time, I can’t do anything else in that moment either.” (Madeleine, 29 years, hetero, TS > 20) |
|  | Partner experiences worries about pregnancy | "[…] ich nehme es oft als Scherz, ich bin so: 'Oh, vielleicht bin ich schwanger!' Und meinen Freund stresst es dann schon sehr, aber ich finde es eigentlich eher lustig. Aber ich glaube auch nicht, dass es schön ist, wenn jemand so irregulär und unvorhersehbar ist." (Flora, 28 Jahre, hetero, TS < 20) | „I often take it as a joke, I’m like, ‘Oh, maybe I’m pregnant!’ And that then really stresses my boyfriend, but I actually find it funny. But I don’t think it’s that nice when somebody is so irregular and unpredictable.” (Flora, 28 years, hetero, TS < 20) |
| Unfavourable influences on one's own well-being | Struggles with managing symptom-related restrictions (e.g., self-blame) | "Und da fühle ich mich dann einfach ein bisschen so - wertlos jetzt nicht, aber jetzt halt auch nicht als vollwertiger Partner, weil ich mir dann einfach so denke: Jetzt sieche ich da wieder herum, zu Hause, und das Leben zieht jetzt nicht vorbei in einer Woche, aber es ist trotzdem einfach ein bisschen eingeschränkter." (Anna, 27 Jahre, hetero, TS > 20) | „And that’s when I simply feel a little like - not worthless, but also not like a worthy partner either because I just think, ‘Now I’m wasting away again, at home,’ and life doesn’t exactly pass by in a week, but it is still just a bit more restricted.” (Anna, 27 years, hetero, TS > 20) |
|  | Difficulties with decisions or evaluating situations | "Wie sind meine Hormone momentan? Möchte ich tatsächlich diese Entscheidung treffen, diese wichtige Entscheidung treffen, oder ist es nur wegen meiner hormonellen Schwankungen? Das beeinträchtigt mich schon." (Franziska, 25 Jahre, feste, TS > 20) | „What are my hormones like right now? Do I genuinely want to make this decision, make this important decision, or is it just because of my hormonal changes? That does affect me.” (Franziska, 25 years, committed, TS > 20) |
|  | Difficulties with self-esteem and sense of security | "Ich fühle mich kaputt, wie nutzlos. Okay, und ich diese extrem dumme Idee, dass ich mir denke, ich muss nutzvoll sein um geliebt zu werden. Was nicht stimmt. Aber trotzdem, das ist so ein Gefühl. Und ich fühle mich nutzlos. […] das bespreche ich auch in der Therapie sehr oft, das: 'Ja, wofür ist eine Frau gut?' Ich denke nicht so. Und ich würde auch nicht so etwas jemandem jemals sagen. Aber so wurde es mir beigebracht. Das ist ein gelerntes, dummes Gefühl. [...] ich will gar keine Kinder [...], aber wenn ich jemals Kinder -wie beschreibe ich das? Das ist crazy. Ich habe nicht die Möglichkeit, [...] also schon, man kann mit Zyklusbeschwerden schwanger werden, aber es ist schwieriger. Ich denke mir so, ich fühle mich nicht wie eine vollständige Frau. Obwohl das Frau-Sein nicht von einem Zyklus abhängig ist. [...] Ich fühle mich weniger eine Frau im Vergleich zu anderen Frauen" (Zoe, 27 Jahre, hetero, TS < 20) | „I feel broken, like useless. Alright, and I [have] this extremely stupid idea that I think that I need to be useful to be loved. Which isn’t true. But still, it’s a kind of feeling. And I feel useless. […] I also discuss it in therapy a lot too, that “What’s a woman good for?” I don’t think like that. And I would never say something like that to anyone either. But that’s what I was taught. It’s a learned, stupid feeling. […] I don’t want any kids […], but if I ever - how do I describe that? It’s crazy. I don’t have the option, […] well kind of, you can get pregnant with menstrual symptoms, but it is more difficult. I personally think that I don’t feel like a whole woman. Although being a woman doesn’t depend on the menstrual cycle. […] I feel less like a woman in comparison to other women. (Zoe, 27 years, hetero, TS < 20) |
|  | Difficulties with setting boundaries | "Also ich merke, ich bin unglaublich empfindlich und kann ganz schwierig auf Frust oder Ablehnung oder wenn mir was unsensibel vorkommt, dann kann das ganz schwer aushalten, ohne dass mich das trifft. Also kann mich da schwer abgrenzen und bin dann einfach leicht traurig oder sauer oder frustriert." (Emma, 26 Jahre, hetero, TS > 20) | „Well, I am noticing that I am incredibly sensitive and I have real trouble with coping with frustration or rejection or if something seems insensitive to me without it affecting me. So, I struggle with distancing myself from that and I then easily get a little upset or annoyed or frustrated.” (Emma, 26 years, hetero, TS > 20) |
|  | Negative associations with other cycle- and relationship-related topics (e.g., miscarriage, desire for children, infertility) | "[...] beeinflusst sich insofern, als dass dieses Thema mit dem Thema Kinderwunsch in Verbindung gebracht werden kann, was für uns kein einfaches Thema war, weil diese OP nach einer Eileiter-Schwangerschaft notwendig war und das mit sehr negativen Gefühlen alles behaftet ist. [...] Und Zyklus bringt diese Erinnerungen wieder zurück, auch was Fehlgeburten angeht und dergleichen Dinge. Also es ist kein neutrales Thema." (Elisabeth, 45 Jahre, hetero, TS < 20) | „[…]has an influence in so far as that the topic can be link to the topic of wanting a child, which wasn’t an easy topic for us as an operation was necessary after the ectopic pregnancy and it’s all associated with very negative feelings. […] And the cycle brings these memories back again, also with regard to miscarriages and the like. So it’s not a neutral topic.” (Elisabeth, 45 years, hetero, TS < 20) |
|  | Worries about negative changes of or within the romantic relationship due to menstrual distress (e.g., changed perception by partner) | "Also meine Laune, oft […] durch den Zyklus hervorgerufen, beeinflusst auf jeden Fall viel […] durch den Zyklus unglaublich irritiert bin und einfach immer zu allem Nein sage oder wütend bin, oder genervt oder gelangweilt. Und das kommt natürlich, weil es ist natürlich nicht schön, wenn man mit der Person dann Zeit verbringt. Und ich würde sagen [...], dass das eben wirklich manchmal Einfluss hat, auf die Meinung von meinem Freund auf mich." (Flora, 28 Jahre, hetero, TS < 20) | „So my mood, which is often […] caused by the menstrual cycle, definitely influences a lot […] as a result of the cycle [I] am incredibly irritated and just always say no to everything or I’m angry or annoyed or bored. And that comes naturally, because of course it’s not nice when you’re spending time with the person. And I would say […] that that genuinely sometimes has an influence, on my boyfriend’s opinion of me.” (Flora, 28 years, hetero, TS < 20) |
|  | Jealousy (e.g., due to physical insecurities) | "Also jetzt gerade ist es sehr stabil. Ich glaube, es liegt auch genau daran, dass ich hier dran gearbeitet habe und nicht mehr so impulsiv bin oder nicht so viele Dinge aufbringe, was, denke ich auch am Zyklus liegen kann. Also dass ich früher sehr, sehr eifersüchtig war, oder irgendwelche alten Sachen, die mal passiert sind, die mich verletzt hatten, dann wieder aufgebracht habe und irgendwie voll viel Wut verspürt habe dem entgegen, die ich gar nicht kontrollieren konnte." (Lena, 23 Jahre, queer, TS > 20) | „Well right now it is very stable. I believe it’s down to precisely the fact that I have worked on it and am no longer as impulsive or bring up as many things, which I think can also be down to the menstrual cycle. So that in the past I was really, really jealous or then I brought old things that had happened and hurt me back up and kind of felt so much anger towards him which I couldn’t control at all.” (Lena, 23 years, queer, TS > 20) |
| Unfavourable influences arising along with a homosexual relationship | Simultaneous cycles: Mutual symptom intensification | "Ja, wir denken beide nicht sehr positiv darüber, also können wir schon mal ein bisschen abrutschen in uns nur darüber beschweren." (Tessa, 19 Jahre, queer, TS > 20) | „Yes, we both don’t view in an especially positive light so we can sometimes drift into only complaining about it.” (Tessa, 19 years, queer, TS > 20) |
|  | Simultaneous cycles: Lack of energy to provide support | "[…] und nicht dann eben auch viel Kraft haben oder sowas, um uns eben darum zu kümmern, ordentlich. Aber sonst helfen wir uns immer ordentlich durch und vor allem, wenn wir nicht gerade beide Schmerzen haben, dann wird immer viel unterstützt." (Tessa, 19 Jahre, queer, TS > 20) | „[…]and then not having much energy or anything to properly deal with it either. But apart from that we always help each other and especially when we’re not currently both in pain, a lot of support is given.” (Tessa, 19 years, queer, TS > 20) |
|  | Alternating cycles: Negative influence on the partner's cycle | "In meiner Beziehung ist es eher so, dass sich mein Partner darüber aufregt, dass ich ihnen die Periode immer weitergeben, weil ich es meistens doch zuerst haben von uns beiden." (Tessa, 19 Jahre, queer, TS > 20) | „In my relationship it’s more like, my partner complains that I always pass my period on to them as I mostly get it first out of the two of us." (Tessa, 19 years, queer, TS > 20) |
| Restrictions of intimacy | Restricted intimacy due to physical factors (pain, libido) | "Also ich würde sagen, […] prämenstruell ist es bei mir eher die Ovulation, wo ich mich wirklich unwohl fühlen kann und eigentlich überhaupt kein sexuelles Verlangen habe. Bei mir ist es andersherum, dass es während der Periode oft besser ist, deswegen ist es da nicht wirklich als Beschwerde gelistet, sondern eher andersherum. Und da ist es natürlich auch, wenn der Zyklus dann sehr irregulär ist oder wirklich ganz komisch ist, dann habe ich manchmal wirklich ganz lange auch kein Verlangen. Das kennt auch jeder, dass es dann manchmal nicht so schön für den Partner ist und auch für einen selbst." (Flora, 28 Jahre, hetero, TS < 20) | „So I would say […] with me it tends to be ovulation premenstrually, when I feel really uncomfortable and actually don’t have any sexual desire at all. With me it’s the other way round, during my period is often better, that’s why it isn’t really listed as a symptom there, but rather more the other way round. And of course, that’s where, when the menstrual cycle is irregular or really completely strange, I then sometimes don’t have any desire for a long time as well. Everyone knows that it’s sometimes not so nice for neither the partner nor oneself.” (Flora, 28 years, hetero, TS < 20) |
|  | Restricted intimacy due to mental factors (ingrained thought patterns, unpleasant feelings) | "Also da denke ich wahrscheinlich schon sehr konservativ, dass ich überhaupt, wenn ich die Periode habe, dass ich da so unrein bin, obwohl ich da nicht so fühle, dass da wahrscheinlich selbstverständlich ist, dass man da nichts bekommt, weder emotional noch körperlich." (Jutta, 43 Jahre, hetero, TS < 20) | „So that’s where I’m probably thinking in a very conservative way that I’m not at all, that I’m so unclean when I’m on my period, even though I don’t feel like it’s self-evident then that you won’t get anything, neither emotionally nor physically.” (Jutta, 43 years, hetero, TS < 20) |
|  | Restricted intimacy due to practical factors (effort, time constraints) | "[…] ich bin grundsätzlich ein Mensch/ Also das Sexualleben ist auf Pause, also eigentlich schon, wenn ich anfange zu prämenstruieren. Bis es dann eben vorbei ist […] Also mir ist es unangenehm. Ich habe auch keine Lust darauf. Das respektiert er total. Aber natürlich beeinflusst es. Die Zeit, wo es dann möglich ist, ist natürlich kleiner, als wenn es anders wäre." (Viktoria , 30 Jahre, hetero, TS > 20) | „[…]in general, I’m a person, well my sex life is technically on hold when I start PMSing. Until it’s over […] So its uncomfortable for me. I’m not in the mood for it either. He totally respects that. But of course it has an influence. The period of time during which it’s possible is obviously smaller than if things were different.” (Viktoria , 30 years, hetero, TS > 20) |
| Unfavourable influences through interaction patterns | Interaction of negative mood (e.g., transference, projection) | "Aber ich merke auch, dass sich so meine Genervtheit über meinen Zyklus so ein bisschen auf ihn überträgt." (Mia, 27 Jahre, hetero, TS > 20) | „But I also notice that my annoyance about my menstrual cycle kind of gets transferred to him.” (Mia, 27 years, hetero, TS > 20) |
|  | Manifestation of negative mood (e.g., changed behaviour, strong emotions) | "Ich würde sagen, es ist ja nicht nur Traurigkeit, sondern vielleicht auch so, dass man eher gereizt ist. Also so, ich weiß, ich zerdenke mir oft so Situationen, ich habe jetzt nicht wirklich Wutausbrüche, aber dass mir das so vorkommt, als würde ich da irgendwie leichter auf etwas gereizt reagieren, wo ich vielleicht sonst eher weniger gereizt oder gar nicht gereizt darauf reagieren würde. Dass mich Sachen einfach schneller aufregen oder ich dann vielleicht schneller traurig werde, obwohl das eigentlich jetzt gar keine großen Sachen eigentlich sind." (Linda, 22 Jahre, hetero, TS < 20) | „I would say it’s not just sadness, but maybe also that you’re more irritable. So like that, I know, I often overthink situations, I don’t really have tantrums, but it then seems to me like I get irritated by things more, which I maybe would have reacted to less irritably or not at all to otherwise. Things just annoy me more or I perhaps get upset more easily, even though they’re technically not a big deal.” (Linda, 22 years, hetero, TS < 20) |
|  | Impairment of shared time (e.g., restricted planning, reduced enjoyment) | "In dem Sinne halt, dass manchmal halt einfach gewisse Pläne einfach ins Wasser fallen oder halt einfach nicht so umgesetzt werden können, wie man es sich vorgenommen hat. Und das ärgert mich dann halt." (Anna, 27 Jahre, hetero, TS > 20) | „In the sense that sometimes certain plans just fall through occasionally or just can’t be carried out the way you’d planned. And that then annoys me.” Anna, 27 years, hetero, TS > 20) |
|  | Changes in relationship dynamics (dependency, reduced sense of teamwork) | "Ich würde sagen, in der Kommunikation. […] Ich würde sagen, meine Reaktionen sind anders, wenn ich gerade von Schmerzen geplagt bin und irgendwie alles gerade chaotisch und emotional einfach komplett durch den Wind bin. Und dann vielleicht antworte ich ungeduldiger, oder vielleicht bin ich dann so: ' Oh, das hast du jetzt gar nicht so gemeint', und interpretiere da Sachen rein, die vielleicht gar nicht da wären." (Pauline, 21 Jahre, hetero, TS > 20) | „I would say in the form of communication. […] I would say that my reactions are different when I’m currently suffering from pain and everything is kind of chaotic and emotionally upside down. And then maybe I respond less patiently or perhaps I’m then like, ‘Oh, you didn’t mean it that way at all,’ and read things into it that weren’t there at all.” (Pauline, 21 years, hetero, TS > 20) |
|  | Reduced quality time due to need for distance | "Ja, also, Schmerz ist ein "Greif mich nicht an", das ist so, also […] man will sich da immer irgendwie schützen, und […] ich bin halt der Typ, ich baue eine Mauer auf und denke mir 'Nein, besser für mich alleine', weil vielleicht bin ich dann irgendwie aggro [...] und das will ich nicht, und dann komme ich in die Vermeidung [...] und ich ziehe mich dann leider ein bisschen zurück." (Tanja, 41 Jahre, hetero, TS < 20) | „Yes, so pain is like ‘don’t touch me,’ it’s so, well […] you always want to protect oneself in a way and […] I happen to be the type of person, I build up walls and think to myself, “nope, I’m better off alone,” maybe because I’m a bit aggressive […] and I don’t want that, and then I slip into avoidance […] and sadly I withdraw a little.” (Tanja, 41 years, hetero, TS < 20) |
| Arising conflicts | Conflicts and conflict escalation due to symptoms | "Naja -- schon beeinträchtigend, weil man […] nimmt dann alles so ernst oder man ist dann einfach grantig und es wirkt sich dann halt auch auf die Bezugspersonen aus, die dann eigentlich überhaupt nichts dafürkönnen." (Sabine, 59 Jahre, hetero, TS > 20) | „Well - kind of impairing because you […] take everything so seriously or you’re just in a huff then and it then has an impact on those close to you too, though it’s not actually their fault.” (Sabine, 59 years, hetero, TS > 20) |
|  | Misunderstandings caused by symptoms or lack of knowledge | "[…] und wenn man dann selbst das Gefühl hat, man kann nichts tun und der andere […] gerade auch nicht adäquat reagieren kann, […] dann […] kann das zu Missverständnissen führen" (Elisabeth, 45 Jahre, hetero, TS < 20) | „[…] and when you have the feeling yourself that you can’t do anything and the other person […] just can’t react adequately either, […] then […] it can lead to misunderstandings.” (Elisabeth, 45 years, hetero, TS < 20) |

## Additional contextual factorsidentified while coding

| **Main Category** | **Associated Category** | **Quote** | **Translation** |
| --- | --- | --- | --- |
| Circumstances within the social environment | Exchange or opportunities for exchange | "Ja, ich habe noch eine sehr, sehr enge Freundin, die neben [Name des Partners] meine zweite Hauptbezugsperson ist, und mit der würde sagen, teile ich noch mehr als mit ihm. Und bei ihr zögere ich zum Beispiel auch nicht, ihr, egal welche Beschwerde mitzuteilen. Da überlege ich gar nicht, ob ich das erzähle oder nicht, weil das teile ich einfach, ungefiltert, und egal, ob das relevant für sie ist oder nicht." (Emma, 26 Jahre, hetero, TS > 20) | „Yes, I have another very, very close friend, who is, along with my boyfriend, my second key support person. And I would say that with her, I share even more than with him. And with her I don‘t hesitate for example, no matter what the symptom. I don‘t consider whether I should tell her or not at all because I just share it, unfiltered, and no matter whether it‘s relevant to her or not.” (Emma, 26 years, hetero, TS > 20) |
|  | Shared experiences | "Früher war das einfach meine Mama, weil, das war meine Bezugsperson Nummer eins. Von da habe ich auch einfach viel Tipps und irgendwie im Umgang bekommen. Und weil ich leider Gottes sehr nach ihr komme, sie hat das ziemlich so wie ich." (Felicitas, 30 Jahre, hetero, TS > 20) | „It used to just be my mum because she was my number one go-to-person. I also just go a lot of tips and a way of handling it from [her]. And unfortunately because I’m very similar to her, she has a similar experience to me with it.” (Felicitas, 30 years, hetero, TS > 20) |
|  | Support and team spirit | "Ja, und das war synchronisiert irgendwie. Und wir haben immer gesagt, zum Beispiel: 'Oh, it's time!' […] Und das war toll, das war das erste Mal in meinem Leben, denke ich, wo ich Zugang hatte zu einer anderen Person, die auch mir, keine Ahnung, es hat sich wie ein Dings gefühlt. Wir sind ein Team. Und das war toll." (Franziska, 25 Jahre, feste, TS > 20) | „Yes, and that was kind of in sync. And we always said, for instance, ‘Oh, it’s time!’ […] And that was great, that was the first time in my life, I think, that I had access to another person who also, I don’t know, it felt like a whole thing. We are a team. And that was great.” (Franziska, 25 years, committed, TS > 20) |
|  | External circumstances | "Ich habe einen Job, wo ich auswählen kann, ob ich im Homeoffice arbeite oder in die Arbeit gehe und die Arbeit ist auch recht nah von mir. Es sind so zehn Minuten Fußweg […]" (Mia, 27 Jahre, hetero, TS > 20) | „I have a job that allows me to choose whether I am working in home office or go into work and my work’s also quite close to me. It’s about a ten-minute walk.” (Mia, 27 years, hetero, TS > 20) |
| Person-related factors | Character and (communication) skills | "Also ich bin halt […] auch anders gestrickt tatsächlich. Ich bin halt vom Wesen her so ein Typ, dass ich halt irgendwie einfach durchziehe. Bis es halt wirklich nicht mehr geht. Und von dem her habe ich es einfach sehr lange überspielt. Und habe mir dann eben gesagt: Das ist jetzt nicht so tragisch oder so. Bis er dann tatsächlich nicht mehr geht." (Anna, 27 Jahre, hetero, TS > 20) | “Well, I’m just ... wired differently, actually. I’m the type of person who just pushes through somehow. Until it really isn’t possible anymore. And because of that, I just played it down for a long time. And at that point I kept telling myself: This isn’t that serious or anything. Until it really wasn’t possible anymore.” Anna, 27 years, hetero, TS > 20) |
|  | Worries and fears (e.g., worsening of symptoms) | "[…] die Angst ist da, dass ich irgendeine Krankheit entwickle, die mit dem Zyklus zusammenhängt. Also ob es Endometriose ist oder so, ich weiß aber nicht, wo der Onset von der Krankheit ist. […] ich bin mir ziemlich sicher, dass aktuell nichts besteht, weil meine Beschwerden nicht so heftig sind [...]. Aber mit der extrem hohen Häufigkeit von Endometriose habe ich schon Angst, dass das entstehen könnte, oder dass sich das noch entwickelt." (Emma, 26 Jahre, hetero, TS > 20) | „[…] the fear is there that I develop some kind of disease that is connected to the menstrual cycle. So, whether it’s endometriosis or something, but I don’t know when the disease’s onset it. […] I am pretty sure that I currently don’t have anything because my symptoms aren’t that bad at the moment […] But considering the extremely high prevalence of endometriosis, I am scared that it could develop or that it will still develop.” (Emma, 26 years, hetero, TS > 20) |
|  | Understanding, engagement, interest in the topic | "Und in den letzten Jahren habe ich mich insofern noch mal intensiver damit beschäftigt, dass es für mich ein politisches Thema geworden ist und ein feministisches Thema. Und deswegen ist es nochmal mehr in den Fokus gerückt." (Emma, 26 Jahre, hetero, TS > 20) | „And in the last years I have occupied myself with the topic more intensely in so far as it has become a political topic and a feminist topic to me. And that’s why it became a greater focus.” (Emma, 26 years, hetero, TS > 20) |
|  | General way of dealing with distress | "Ich glaube, ich war sehr streng zu mir selbst eine Zeit lang, also wahrscheinlich bis zur Mitte des Studiums. War ich so: Okay, ich musste Sachen machen und so weiter. Irgendwann habe ich es mir dann einfach rausgenommen, Pläne anders zu gestalten, mir auch diese Flexibilität selbst zu geben. Wenn ich etwas ausmache, sage ich meistens gleich dazu, es kann sein, dass ich da in den Tagen meine Tage bekomme. Und es kann dann auch sein, dass ich spontan absage und versuche, das immer sehr offen zu kommunizieren, damit ich keinen Stress habe, das dann abzusagen, obwohl es mir nicht gut geht und ich in diesen Konflikt nicht gerate." (Kim, 27 Jahre, hetero, TS > 20) | „I think I was very strict with myself for a time, like probably until the middle of my degree. Was like: Okay, I had to do things and so on. At some point I just gave myself the right to plan things differently, to give myself this flexibility as well. When I agree to something, I usually immediately add that it’s possible that I’ll get my period then. And it’s then also possible that I spontaneously cancel and I try to always communicate that very openly so that I don’t have any stress about cancelling it even though I’m not feeling well, and I don’t end up in that conflict.” (Kim, 27 years, hetero, TS > 20) |
|  | Overall attitude (e.g., acceptance of body and cycle) | "Ich mag es, meine Menstruation zu bekommen. Ich freue mich, wenn ich sie bekomme. Auch generell, auch aus gesundheitlicher Perspektive. Ich finde es wichtig für eine Frau, dass man sie regelmäßig hat. Ich habe nur Positives in Verbindung." (Lina, 24 Jahre, hetero, TS > 20) | „I like getting my period. I’m glad when it arrives. Also just in general, from a health perspective as well. I find it important for a woman to regularly have it. I only have positive associations.” (Lina, 24 years, hetero, TS > 20) |
| Cycle-related factors | Previous experiences with menstrual distress (including contrast effects) | "Da sich die Zyklusbeschwerden erst innerhalb der Partnerschaft entwickelt haben, weiß ich jetzt nicht, wie die Partnerschaft meine Zyklusbeschwerden beeinflussen." (Elisabeth, 45 Jahre, hetero, TS < 20) | „As my menstrual symptoms only developed during the relationship I don’t know how my relationship influences my menstrual symptoms.” (Elisabeth, 45 years, hetero, TS < 20) |
|  | Interaction of menstrual distress with other factors | "Ich habe vor allem auch chronische Rückenschmerzen, und […], wenn ich Pech habe, wird es dann vor allem in den Phasen schlimm, und wenn dann irgendwie die Blutung sozusagen aktiv ist in den paar Stunden, dann kann es sehr, sehr wehtun, dass mein unterer Rücken sich wirklich anfühlt, als würde er in zwei gebrochen werden [...]" (Flora, 28 Jahre, hetero, TS < 20) | „I also have chronic back pain in particular and […], if I’m unlucky, it gets bad in those phases especially, and if the bleeding is ongoing in those few hours, then it can really, really hurt, so that my lower back really feels like it’s being broken in two.” (Flora, 28 years, hetero, TS < 20) |
|  | Course of the cycle (including awareness and predictability) | "Ich glaube, damals habe ich noch viel länger gebraucht, um zu realisieren, dass es an meinem Zyklus liegt, dass es mir schlecht geht und dass es nicht an anderen Sachen liegt. Aber ich weiß, damals, ich habe immer Tagebuch geschrieben und ich habe es immer gemacht, als es mir schlecht ging und irgendwann habe ich gemerkt: 'Aha!' Es ist echt in einem so regelmäßigen Monatszyklus, dass ich das mache und dass es eben auch mit meinem Zyklus übereinstimmt." (Mia, 27 Jahre, hetero, TS > 20) | „I think I took a lot longer to realise that it was down to my menstrual cycle that I was feeling bad and that it wasn’t down to other things. But I know back then I always kept a diary and I always wrote in it when I felt rough and at some point I noticed, ‘Aha!’ I really do it in a regular monthly cycle and it happens to match my menstrual cycle as well.” (Mia, 27 years, hetero, TS > 20) |
|  | Symptoms and level of distress | "Ich bin irgendwann mal ins Krankenhaus gegangen, weil ich dachte, es wird eine Blinddarmentzündung. Aber es war nur eine Zyste, die geplatzt ist, also das war es nicht." (Madeleine, 29 Jahre, hetero, TS > 20) | „At some point I went to hospital because I thought I had appendicitis. But it was just a cyst that had burst, so it wasn’t that.” (Madeleine, 29 years, hetero, TS > 20) |
|  | Functionality in daily life | "Und jetzt ist es wirklich so, dass ich […] Alltagstätigkeiten nicht mehr nachkommen kann. Oder nicht zu Fuß gehen kann oder solche Sachen. Insofern ist es so stark einschränkend […]" (Elisabeth, 45 Jahre, hetero, TS < 20) | „And now it’s really at the point where I […] can’t stay on top of everyday tasks. Or can’t go to places on foot or things like that. In that regard it is extremely debilitating.” (Elisabeth, 45 years, hetero, TS < 20) |
|  | Medication and compensation | "Also das sage ich dann nicht ab, das sind dann eher private Sachen, wenn ich Arbeit habe oder so, werfe ich mir eine Schmerztablette rein" (Julia, 28 Jahre, hetero, TS > 20) | „So, I don’t cancel that, that’s more the case with private things, when I have work or something I just take a painkiller.” (Julia, 28 years, hetero, TS > 20) |
| Circumstances within the relationship | Formal aspects (duration, cohabitation, type) | "Und seitdem wir zusammenwohnen, bekommt er halt auch den ganzen Zyklus mit und hat auch irgendwie angemerkt, dass er das voll krass findet, dass es so anders ist. Also, dass ich irgendwie dann auch mal dadurch, dass ich so nah mit einer Person zusammenwohne, und in einer WG, wo ich vorher gewohnt habe, ist es halt nicht so nah, dass die Leute das so krass mitbekommen." (Lena, 23 Jahre, queer, TS > 20) | „And since we’ve been living together, he witnesses the whole cycle and he sort of commented that he finds it really crazy that it’s so different. So I then kind of also as a result of the fact that I’m living so close with another person, and in a shared flat, like I used to live in, it’s just not close enough that people really notice it.” (Lena, 23 years, queer, TS > 20) |
|  | Communication culture (general and cycle-related) | "Also es gibt nichts, was ich nicht ansprechen würde, oder mich nicht wohlfühlen würde, anzusprechen." (Madeleine, 29 Jahre, hetero, TS > 20) | „So, there is nothing that I wouldn’t bring up or wouldn’t feel comfortable mentioning.” (Madeleine, 29 years, hetero, TS > 20) |
|  | Growing together (including overcoming other difficulties) | "Mittlerweile sind wir auch über verschiedene Hürden gemeinsam durch. Einmal war es in seiner Familie, dass jemand sehr krank war, dann war es in meiner Familie, dass jemand sehr krank war. Diese Dinge, wo man sich gegenseitig auch unterstützt, das ist dann auch etwas, das stärkt zum Glück in unserem Fall die Beziehung." (Madeleine, 29 Jahre, hetero, TS > 20) | „In the meantime, we’ve also made it through various hurdles together. One time somebody was sick in his family, then there was someone sick in my family. These things that you support each other with, they are things that fortunately strengthen the relationship in our case.” (Madeleine, 29 years, hetero, TS > 20) |
|  | Informal aspects (seriousness, balance, teamwork) | "Wir unterstützen uns, ich unterstütze ihn auch. Aber eben, in den letzten fünf Monaten habe ich ein bisschen mehr Unterstützung gebraucht." (Zoe, 27 Jahre, hetero, TS < 20) | „We support each other, I support him to. But in the last five months I needed a bit more support.” (Zoe, 27 years, hetero, TS < 20) |
|  | Inherent feelings (e.g., love, trust, understanding, security) | "Und eigentlich funktioniert es auch sehr gut seit vier Jahren. Es ist sehr, ich weiß nicht, ob das jetzt so romantisch klingt, aber es ist sehr stabil, alles. Es funktioniert sehr gut und es hat sich seit vier Jahren, ehrlich gesagt, nicht viel geändert, was ganz angenehm ist. [...] im Sinne von: Es schwankt nicht die ganze Zeit irgendwie rauf und runter. Es ist sehr: Man kann sich darauf verlassen." (Helena, 20 Jahre, queer, TS < 20) | „And it’s actually been working really well for four years. It is very, I don’t know whether this exactly sounds romantic, but it very stable all round. It works very well and to be honest, not much has changed in the last four years which is quite comfortable. […] In the sense of: It’s not sort of fluctuating all the time. It’s very much: You can rely on it.” (Helena, 20 years, queer, TS < 20) |
|  | General conflict level (independent of menstrual distress) | "Leider -- ein bisschen -- nicht repräsentativ, weil wir haben eine bisschen schwierige Zeit hinter uns. Deswegen war es schon mit sehr viel Anstrengung und emotionaler Arbeit und mit sehr viel Konflikt verbunden. Das hat auch ein bisschen zu Unsicherheiten geführt. Deswegen -- in den letzten drei Monaten war es eher ein bisschen unsicher und anstrengend. Aber natürlich nicht nur. Und wir hatten auch trotzdem sehr schöne Momente" (Emma, 26 Jahre, hetero, TS > 20) | „Unfortunately not quite representative, because we’ve gone through a bit of a difficult time. That’s why it was already associated with a lot of strain and emotional labour and with a lot of conflict. That also kind of led to uncertainties. That’s way, in the last three months it was sort of uncertain and strenuous. But of course not just that. And we still had beautiful moments too.” (Emma, 26 years, hetero, TS > 20) |
|  | Shared daily life (contact, time, interests, stress, activities) | "Das hat damit zu tun, dass seine Zeit so beschränkt ist, also die Zeit für uns oder überhaupt für unsere Partnerschaft." (Jutta, 43 Jahre, hetero, TS < 20) | „That’s to do with the fact that his time is so limited, so [his] time for us or for our relationship in the first place.” (Jutta, 43 years, hetero, TS < 20) |
|  | Personal aspects of the partner (e.g., personality/nature, knowledge, interest, reactions) | "Er studiert auch Medizin. Generell interessiert es ihn. Voll. Und nachdem er eine Freundin bekommen hat, hat es ihn erst recht interessiert." (Lina, 24 Jahre, hetero, TS > 20) | „He’s studying medicine. It interests him in general. A lot. And once he got a girlfriend, it really interested him.” (Lina, 24 years, hetero, TS > 20) |
| Societal factors | Perceived norm (e.g., allowed to show symptoms or make inconveniences) | "Ich meine, es passiert eh schon ein bisschen in der Gesellschaft, dass es nicht mehr ganz so ein Tabuthema ist, aber es ist immer noch ein Ding von: Wenn man als Frau Zyklusbeschwerden hat, oder die Blutung. Dann ist es ein Ding von: muss sich die Person wieder zurückziehen." (Felicitas, 30 Jahre, hetero, TS > 20) | “I mean, it’s already changing in society to be less of a taboo topic anyway, but it’s still always a case of if you as a woman are experiencing menstrual symptoms or you’re on a period, then it’s a case of the person having to hide away again.” (Felicitas, 30 years, hetero, TS > 20) |
|  | Perceived importance of the topic | "Und ich denke mal, natürlich habe ich auch Beschwerden während der Menstruation, aber irgendwie ist das so am normalsten oder vielleicht auch sehr normalisiert von der Gesellschaft, dass ich das irgendwie so hinnehmen kann." (Klara, 24 Jahre, hetero, TS > 20) | „And I think, of course I have symptoms during my period, but somehow that’s the most normal or perhaps so normalised by society that I can kind of just accept it.” (Klara, 24 years, hetero, TS > 20) |
|  | Destigmatisation and normalization (vs. stereotyping) | "Und mir ist das dann schon wichtig, […] dass das auch besprochen wird, weil es mich vor allem auch immer extrem belastet, dass das ein Thema ist, das so totgeschwiegen wird und wo auch verlangt wird, dass man das wegsteckt und nicht wirklich darüber redet." (Dana, 21 Jahre, hetero, TS > 20) | „And it is quite important to me […] that it also gets discussed because it always extremely stresses me that it’s a topic that is hushed up and where there’s also a requirement for you to put it away and not really talk about it.” (Dana, 21 years, hetero, TS > 20) |
|  | Fear of stereotyping | "Manchmal sprechen wir darüber, aber ich weiß auch nicht, wie ich dazu stehe, manchmal zu sagen: 'Das ist gerade, weil ich meine Tage bekomme'. Weil: Ich habe das Gefühl, es klingt manchmal wie eine Rechtfertigung, wenn man jetzt so eine Emotion hat, und die ist halt vielleicht dadurch produziert, weil der Körper hormonell gerade arbeitet [...]. Ich es nicht erklären, aber es ist für mich so ein bisschen dieses Stereotyp, man hat voll die Stimmungsschwankungen, weil man jetzt seine Tage bekommt oder seine Tage hat." (Marie, 24 Jahre, hetero, TS > 20) | „Sometimes we talk about it, but I also don’t know how I feel about sometimes saying, ‘This is happening right now because I’m on my period.’ Because I have the feeling that it sometimes sounds like a justification if you’re experiencing an emotion, and it’s maybe produced by the fact that your body is currently hormonally active […] I [can’t] explain it, but for me it’s kind of that stereotype of having major mood swings because you’re getting your period or are on your period.” (Marie, 24 years, hetero, TS > 20) |
|  | Reference framework (comparison and evaluation) | "Also meine Schwester vielleicht ein bisschen, weil sie halt Endometriose hat, und das auch erst vor zwei, drei Jahren diagnostiziert bekommen hat, und davor halt krasse Schmerzen hatte, einfach. Und ich glaube, ich habe mich immer ein bisschen damit verglichen, weil so: 'Oh mein Gott, ich kotze ja nicht, also so schlimm kann es nicht sein irgendwie. Nur dass das halt absolut kein Maßstab ist." (Julia, 28 Jahre, hetero, TS > 20) | „Well maybe my sister a little, because she has endometriosis and also only got it diagnosed two, three years ago and was just in really severe pain beforehand. And I think I always compared myself with that a bit because like, “Oh my god, I’m not vomiting, so it can’t be that bad.” Only that’s really just not a criterion.” (Julia, 28 years, hetero, TS > 20) |
|  | Perceived care structure (resources, sources of information) | "Auch gibt es wenig Anlaufstellen, wo man überhaupt darüber reden kann. Also nicht jeder Frauenarzt ist vielleicht so ein guter Gesprächspartner, manche stehen auch unter Zeitdruck. […] Also mit meinem kann ich nicht so darüber reden, obwohl ich ihn mag und schätze. [...] Aber es gibt von jetzt der Stadt Wien keine Anlaufstellen, wo man darüber reden kann." (Jutta, 43 Jahre, hetero, TS < 20) | „Also, there are so few contact points where you can talk about it in the first place. Like, not every gynaecologist is such a great conversational partner, some are also under time pressure. […] Well, I can’t really talk about it with mine, even though I like and appreciate him. […] But the city of Vienna doesn’t have any points of contact where you can talk about it.” (Jutta, 43 years, hetero, TS < 20) |
|  | Professional contact persons (medicine, psychotherapy, other institutions) | "Ja, es ist so schwierig, weil ich bei jedem Arzt, bei dem ich bis jetzt war, der konnte mir nichts diagnostizieren. Der war so, ja, ich weiß nicht. Und meine Gynäkologin will sich gar nicht anschauen, ob ich Endometriose haben könnte. Es wäre um einiges leichter, wenn das Gesundheitssystem rund darum wesentlich besser gestaltet wäre, glaube ich. Und ich glaube, [...] dann wäre es auch einfacher, damit umzugehen." (Helena, 20 Jahre, queer, TS < 20) | „Yes, it’s so difficult because no doctor I’ve had so far could diagnose anything. He was like, ‘Yes, I don’t know.’ And my gynaecologist doesn’t even want to check whether I could have endometriosis. I believe it would be so much easier if the underlying health system were designed much better. And I think it would then also be better at it, […] then it would also be eaiser to handle it.” (Helena, 20 years, queer, TS < 20) |
